# Convergence without divergence in North American red-flowering *Silene*

**DOI:** 10.3389/fpls.2022.945806

**Published:** 2022-09-06

**Authors:** Andrea E. Berardi, Ana C. Betancourt Morejón, Robin Hopkins

**Affiliations:** ^1^Harvard University Herbaria, Cambridge, MA, United States; ^2^Department of Organismic and Evolutionary Biology, Harvard University, Cambridge, MA, United States; ^3^The Arnold Arboretum, Boston, MA, United States; ^4^Department of Biology, University of Puerto Rico - Rio Piedras Campus, San Juan, Puerto Rico; ^5^Department of Ecology and Evolutionary Biology, Yale University, New Haven, CT, United States

**Keywords:** floral color, floral morphology, convergent evolution, pollination syndrome, *Silene*

## Abstract

Combinations of correlated floral traits have arisen repeatedly across angiosperms through convergent evolution in response to pollinator selection to optimize reproduction. While some plant groups exhibit very distinct combinations of traits adapted to specific pollinators (so-called pollination syndromes), others do not. Determining how floral traits diverge across clades and whether floral traits show predictable correlations in diverse groups of flowering plants is key to determining the extent to which pollinator-mediated selection drives diversification. The North American *Silene* section *Physolychnis* is an ideal group to investigate patterns of floral evolution because it is characterized by the evolution of novel red floral color, extensive floral morphological variation, polyploidy, and exposure to a novel group of pollinators (hummingbirds). We test for correlated patterns of trait evolution that would be consistent with convergent responses to selection in the key floral traits of color and morphology. We also consider both the role of phylogenic distance and geographic overlap in explaining patterns of floral trait variation. Inconsistent with phenotypically divergent pollination syndromes, we find very little clustering of North American *Silene* into distinct floral morphospace. We also find little evidence that phylogenetic history or geographic overlap explains patterns of floral diversity in this group. White- and pink-flowering species show extensive phenotypic diversity but are entirely overlapping in morphological variation. However, red-flowering species have much less phenotypic disparity and cluster tightly in floral morphospace. We find that red-flowering species have evolved floral traits that align with a traditional hummingbird syndrome, but that these trait values overlap with several white and pink species as well. Our findings support the hypothesis that convergent evolution does not always proceed through comparative phenotypic divergence, but possibly through sorting of standing ancestral variation.

## Introduction

Angiosperm evolution is characterized by extensive diversification of floral form and function (Stebbins, 1970). The plant-pollinator relationship has been one of the major drivers of the angiosperm radiation (Stebbins, 1970; Faegri and van der Pijl, 1979) at both microevolutionary and macroevolutionary scales (Van der Niet and Johnson, 2012; Van Der Niet et al., 2014). Selection by particular pollinator groups has resulted in the evolution of convergent combinations of specific floral traits, known as pollination syndromes, across the phylogeny of flowering plants (Faegri and van der Pijl, 1979; Fenster et al., 2004; Schiestl and Johnson, 2013). Flowering plants have evolved traits such as color and scent to attract pollinators, corolla shape, and reproductive organ placement to ensure efficient pollen transfer, and nectar amount and sugar concentration to provide desirable rewards (Grant, 1951). Although there are numerous well-studied examples across angiosperms of closely related species evolving distinct “syndromes” characterized by combinations of floral traits associated with a particular pollinator [e.g., moth, hummingbird, and bee pollination in *Petunia, Mimulus*, and *Aquilegia* (Schemske and Bradshaw, 1999, Stuurman et al., 2004, Whittall and Hodges, 2007, Sheehan et al., 2012)], the extent to which pollinators drive diversity in floral traits is still unknown in many groups of flowering plants.

Different suites of trait values optimize pollination by different types of animals leading to not only correlations between dominant pollinator type and floral traits, but also correlations between genetically independent traits within a flower (Faegri and van der Pijl, 1979; Fenster et al., 2004; Hermann et al., 2013; Schiestl and Johnson, 2013). For example, red flowers tend to have long tubular corollas with exerted reproductive organs and abundant nectar since these traits are associated with efficient hummingbird pollination (Grant, 1951). Hawkmoth pollinated plants tend to have white flowers with long narrow tubular corollas, and strong scents (Grant, 1951). Although bees are diverse, many bee-pollinated plants have purple, pink, or yellow flowers, with wide, open corolla tubes that often form a lip for landing, and nectar guides (Grant, 1951). In many clades of plants, these pollination syndromes have independently evolved numerous times causing strong correlations among floral traits and clusters of phenotypes in morphological space (Wilson et al., 2004; Smith et al., 2008; Van der Niet and Johnson, 2012; Lagomarsino et al., 2017). Yet, in other plant systems, there appears to be extensive variation in floral form that does not match classic pollination syndromes, as well as the evolution of generalized pollination syndromes that attract multiple pollinator groups (Ollerton, 1996; Waser et al., 1996; Muchhala et al., 2009; Dellinger, 2020). Understanding the extent to which convergent evolution has resulted in correlated floral trait diversity across phylogenetic diversity is important for understanding if and how pollinators are driving evolution in different groups of angiosperms.

Floral color is one of the most well-studied floral “syndrome” traits, as it is an important visual cue for both pollinators and researchers. Color is often used for pollinator preference and flower discrimination, which can lead to reproductive isolation and speciation (Bradshaw and Schemske, 2003; Hoballah et al., 2007; Hopkins and Rausher, 2011; Sheehan et al., 2016). In fact, in some macroevolutionary studies of pollination syndrome, assumptions about the association between floral color and pollinator preference have led to predictions about pollination biology largely based on variation in floral color (Lagomarsino et al., 2017). However, floral color is not always the most important trait nor the most reliable indicator of the major pollinator (Smith et al., 2008; Rosas-Guerrero et al., 2014). In some systems, diversity occurs across many highly correlated floral traits and, while color can act as an easy phenotypic cue for identifying pollination syndrome, it is essential to consider the distribution of variation in other floral traits such as morphology, scent, and nectar rewards as they may be equally or more important (Smith et al., 2008; Klahre et al., 2011). Specific floral traits or trait combinations can shape plant-pollinator interactions under different conditions, and often vary greatly among plant taxa.

Patterns of floral trait diversification and phenotypic convergence across a genus can result from the dynamic interaction of many factors including phylogenetic constraint, geographic context, variation in ploidy, and, of course, pollinator-mediated selection. First, related species can share similarities both at the genetic level and the phenotypic level due to shared ancestry. In this way, measured phenotypic variance in traits between species may not be truly independent of each other but instead constrained by common ancestral genetic variation (Uyeda et al., 2018). For this reason, controlling for phylogenetic relatedness is essential in evaluating phenotypic convergence and evolutionary patterns, since traits in related species may be similar through descent from a shared ancestor rather than from shared selection regimes alone.

Second, the degree to which closely related species diverge or maintain similarity may be dependent on the geographic proximity of the two species. Related species that occur in sympatry are predicted to have greater phenotypic divergence than in allopatry due to selection to reduce costly hybridization and decrease competition for a shared resource (such as pollination; Dobzhansky, 1940; Mayr, 1942). Floral morphology and color are key traits that can respond to disruptive selection in sympatry due to competition for pollinators. In many systems, closely related species show both simple and extensive divergence in floral traits when in sympatry but not in allopatry (Grossenbacher and Whittall, 2011; Hopkins and Rausher, 2012; Briscoe Runquist and Moeller, 2014). One example is *Phlox drummondii*, which has blue flower color in allopatric ranges but evolved a red flower color in sympatry with *Phlox cuspidata* due to pollinator-mediated selection (Hopkins and Rausher, 2012). Thus, the degree of geographic overlap across a group of plants may influence the extent to which pollinator-driven selection is disruptive and causes a divergence between closely related species. Convergent and repeated transitions to specific pollinators and syndromes [such as countless transitions to hummingbird pollination in North America; Grant, 1994)] must be carefully considered in the context of both phylogeny and geography.

Third, polyploidy can play an important role in evolution and speciation, and can have direct effects on floral phenotypic variation. Not only can polyploidization events lead to increased rates of speciation, but they can also generate both genetic and phenotypic novelty (Soltis et al., 2014). Importantly, changes in polyploid lineages to floral size, floral morphology, floral scent, and floral color in several plant taxa are often sufficient to attract a separate class of pollinators (Segraves and Thompson, 1999; McCarthy et al., 2016; Palmqvist et al., 2021). The changes induced by polyploidy, through several different genetic mechanisms, can have profound effects on floral signals. Thus, ploidy is important to account for when investigating patterns of floral evolution.

The genus *Silene* (Caryophyllaceae) contains more than 800 species of annual and perennial herbaceous plants, distributed predominantly throughout the Northern Hemisphere (Petri and Oxelman, 2011). While the genus is defined by some key floral traits, such as fused and often inflated calyces, *Silene* are diverse across many characters, including reproductive systems (dioecy, gynodioecy, hermaphroditism, and selfing), ecological specialization (circumpolar species, desert species, alpine and montane species, endemic species, invasive species) and morphological variation (both vegetative and reproductive; Hitchcock and Maguire, 1947; Oxelman et al., 2001; Popp and Oxelman, 2001; Moyle, 2006; Frajman et al., 2018). The North and Central American species of *Silene* [most belonging to section *Physolychnis sensu lato* within subgenus *Behenantha* (Dumort.) Rohrb., age estimate 3.37 MYA based on Sloan et al. (2009)] are particularly variable in floral form among and within species, making for challenging taxonomic treatments based on morphology alone (Hitchcock and Maguire, 1947; Chowdhuri, 1957; Burleigh and Holtsford, 2003; Popp and Oxelman, 2007).

**Figure 1:**
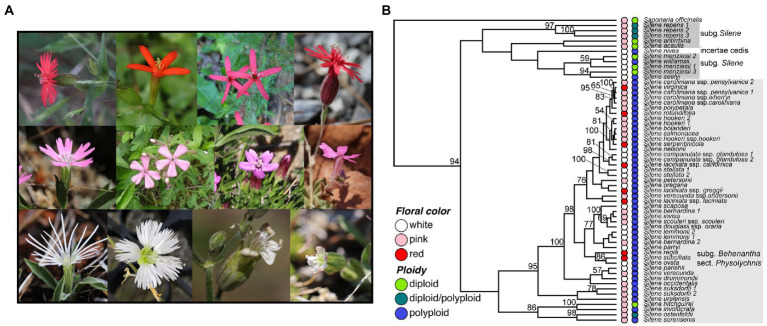
**(A)** Photos of four representative red-, pink-, and white-flowering native North American *Silene* species. Upper panel (red flowers), left to right: *S. laciniata ssp. californica*, *S. subciliata*, *S. virginica*, and *S. serpentinicola*. Middle panel (pink flowers), left to right: *S. hookeri*, *S. caroliniana*, *S. acaulis*, and *S. petersonii*. Lower panel (white flowers), left to right: *S. nelsonii, S. stellata, S. sargentii, and S. parryi*. **(B)** Maximum likelihood ITS gene tree with bootstrap support values demonstrating the distribution of floral color and ploidy in North American *Silene* sect. *Physolychnis* s.l. and other native North American *Silene* (sections boxed in gray)*. Saponaria officinalis* is included as an outgroup, and the tree is scaled using the divergence time between *Saponaria* and *Silene*. Floral color and ploidy are indicated in the colored circles adjacent to taxa. Bootstrap support values > 50 are shown to the left of nodes. Photo credits: *S. laciniata, S. subciliata, S. virginica, S. serpentinicola, S. caroliniana, S. stellata* by A. Berardi; *S. hookeri, S. acaulis, S. petersonii, S. nelsonii, S. sargentii, S. and parryi* by J. Miladin.

Although not all North and Central American species belong to section *Physolychis*, it is of specific interest because it contains the most variation in ploidy and floral color (Figure 1). Most *Physolychnis* are polyploid (ranging from tetraploids 2*n* = 4x = 48 to octoploids 2n = 8x = 72, rarely decaploids 2 N = 10x = 120) with a small number diploid/polyploid variable species (Popp and Oxelman, 2007; Frajman et al., 2018). The polyploid species are likely allopolyploids allowing for any number of possible combinations of parental lineages resulting in extensive phenotypic diversity (Popp and Oxelman, 2007). Of particular note, *Physolychnis* in North and Central America is also the only set of taxa to have evolved red floral color in *Silene sensu stricto* (with the single exception recently placed in *Silene sensu lato*, the orange-red *Lychnis chalcedonica* syn*. Silene chalcedonica* native to Eurasia). Given the frequent pairing of red flower evolution and shifts to hummingbird pollination (Grant, 1994), and the geographically restricted presence of hummingbirds to the Americas, it is hypothesized that novel hummingbird pollination is selected for red flower color evolution in North American *Silene.*

Despite this extensive diversity, only a small subset of species in *Physolychnis* have been phenotypically characterized and very little is known about their pollination biology. The most well-known and well-studied species in this section, *S. virginica*, *S. caroliniana*, and *S. stellata* (Dudash and Fenster, 2001; Fenster et al., 2006; Reynolds and Fenster, 2008; Reynolds et al., 2009), loosely adhere to traditional pollination syndromes and are viewed as “specialized” to hummingbirds, bees, and moths, respectively (Reynolds et al., 2009). The remaining *Silene* native to North America either have no published pollinator observations or are simply presumed to be pollinated by particular pollinator groups; i.e., most red-flowering species are presumed to be pollinated by hummingbirds, but in practice, they are often also visited by butterflies and bee-flies (A. Berardi, personal observation). Whether standard expectations of pollination syndromes apply to *Silene* section *Physolychnis* is unclear.

Given the notable morphological diversity and novel red floral color in North American *Silene*, we used a floral morphospace approach to determine (1) whether species cluster into distinct morphological groups and (2) whether morphological groups cluster by floral color. Toward these goals, we quantify floral trait correlation patterns to infer whether floral color groups converged upon suites of characters. Through our analyses, we take into account phylogenetic and geographic contexts of divergence and ploidy variation. With some exceptions, we find that generally, floral color is not a good indicator for phenotypic convergence and morphological grouping in North and Central American *Silene.*

## Materials and methods

### Trait data collection

We took the medians of the phenotypic ranges recorded in the Flora of North America (FNA) and the Jepson eFlora, except when noted, for the following traits: stem length, leaves per node, leaf length, calyx height, calyx width, and petal limb length. We categorized the following traits: inflorescence (one, few 1–3, many >3), floral tube extension from calyx (below calyx, equal to calyx, and above calyx), reproductive organ exsertion (below corolla, equal to corolla, extended above corolla). Floral color was assigned into three bins: white, pink, and red. *Silene* species that have white/pink floral color polymorphism were generally coded as pink to reflect the ability of the species to produce pigment in their petals. The single species that is a pale yellow, *S. parishii*, was coded as white to reflect the absence of anthocyanin pigments. The orange-pink *S. salmonacea* was coded as pink. Ploidy information was taken from a combination of sources including the FNA and the literature (Kruckeberg, 1954, 1955, 1960, 1961, 1964; Spellenberg, 1979).

Floral characters described in the FNA as ratios were calculated from the median value (e.g., “corolla 11/4 times longer than calyx height”), and were otherwise measured manually or an alternate source was sought. For species and traits that were not included or calculable in the FNA we used other flora, field guides, literature, herbarium measurements, and field measurements, see Supplementary Table 5.

### ITS gene tree estimation

Publicly available ITS (internal transcribed spacer) sequences for most taxa in our dataset were retrieved from Genbank. In total, we gathered 47 sequences for 37 species and recognized subspecies in subgenus *Behenantha* section *Physolychnis*, one sequence for one species in subgenus *Behenantha* incertae sedis, five sequences for three species in subgenus *Silene* section *Anotites*, three sequences for one species in subgenus *Silene* section *Auriculatae*, two sequences for two species in subgenus *Silene* section *Siphonomorpha*, and one sequence for one species in subgenus *Silene* section *Sclerophyllae* (Supplementary Table 5). Additionally, ITS sequences were fetched for outgroup *Saponaria officinalis* and for non-native *Silene nutans* which was added only for the ITS tree supporting the ancestral state reconstructions. ITS sequences were unavailable for *S. nuda, S. plankii, S. sargentii,* and *S. wrightii.* Most ITS sequences were taken from Popp and Oxelman (2007) and Mesler et al. (2020); all accessions used are listed in Supplementary Table 5.

Sequences were aligned with the MAFFT algorithm in Geneious v9.1.8 (Biomatters Ltd.). The sequence alignment was trimmed by eye to a final alignment length of 661 nt. Only partial sequences (ITS2 only) were available for some taxa and are noted in Supplementary Table 5. Because of this, we partitioned the alignment into ITS1 and ITS2 for phylogenetic analysis. We used ModelTest-NG v0.1.7 to estimate the best nucleotide model of evolution with AIC and BIC selection, which was GTR + GAMMA for both partitions (Darriba et al., 2019). Phylogenetic relationships were estimated in RAxML v8.2.11 (Stamatakis, 2014) by estimating the best-scoring maximum likelihood tree with 1,000 bootstrap replicates (using the rapid RAxML bootstrapping algorithm, −f a), and the entire analysis was performed in duplicate. The computations were run on the FASRC Cannon cluster supported by the FAS Division of Science Research Computing Group at Harvard University.

We inferred relative divergence times for the ITS tree to generate an ultrametric tree for downstream phylogenetic PCA analysis. We used the divergence times for Caryophyllaceae described in Frajman et al. (2009) to calibrate the tree (maximum age of 31.7 Myr for Saponaria – Silene divergence and minimum age of 12.39 Myr for the subgenera *Silene* and *Behenantha* in tribe Sileneae that have North American lineages) with the “chronopl” function in the R package ape, with no smoothing parameters (lambda = 0).

### Floral trait correlations, phylogenetic ANOVA, and phylogenetic signal

To look for evidence of tightly correlated floral traits as an indicator of pollination syndromes, we took categorical floral trait characters (tube extension from calyx, reproductive organ extension, petal color, and inflorescence class) and converted them to representative integers (e.g., 1, 2, 3 or −1, 0, 1, described in the section below), and then combined them with quantitative traits (calyx height, calyx width, and petal limb) to perform a correlation analysis. We analyzed floral trait correlations for the entire dataset and for each species subset by floral color, specifically to address whether any floral traits are correlated in North American *Silene* as well as whether specific floral colors are an indicator of specific combinations of correlated floral traits (and thus an indicator of possible pollinator syndromes). We used Spearman’s rank correlations and corrected *p*-values for multiple comparisons using Holm’s method with base R functions. To visualize correlations, we used the R package corrplot [v0.92; Wei and Simko (2021)]. Correlation coefficient values are provided in Supplementary Table 3.

To determine whether any traits (vegetative or floral) are explained by floral color, we used a phylogenetic ANOVA with the R package phytools function *phylANOVA* [v1.0–1, (Revell, 2012)]. For each regression, we provided the ultrametric ITS gene tree and performed 1,000 simulations with *post-hoc t-test*s (corrected for multiple comparisons with the Holm technique). We tested each floral trait for phylogenetic signal using Blomberg’s K and Pagel’s Pagel’s λ using the R phytools function “phylosig” with hypothesis testing (test = T).

### Ancestral state reconstruction and stochastic character mapping

In order to understand whether red floral color arose recently and independently across North American *Silene,* we reconstructed the evolution of floral color using maximum likelihood with the *ace* function in R package ape (Paradis et al., 2004) and stochastic mapping using *make.simmap* in R package phytools (Revell, 2012), both using the ultrametric ITS tree. We tested seven different models of discrete character evolution for floral color (red, pink, and white): all rates different (ARD), equal rates (ER), symmetric rates (SYM), and custom stepwise models stepwise reversible, stepwise pink-red irreversible, stepwise red-white irreversible, and stepwise red-pink and red-white irreversible. Constrained and free parameters for each model are described in Supplementary Table 1. We compared the likelihood and AIC scores for each model and selected the best by the lowest AIC score, the ER model. Since the equal rate model was not more than two AIC scores away from the stepwise reversible model (white ←→ pink ←→ red), we chose to run both models.

Stochastic mapping is a Bayesian approach that simulates possible histories of discrete character evolution on a phylogeny based on a model of character evolution and using the posterior probability distribution (Huelsenbeck et al., 2003). We performed 1,000 simulations of stochastic mapping for discrete character evolution using both the ER and reversible model and fixed the root at equal probabilities for all colors. The simulations were summarized to give the posterior probability of each state at each node, as well as posterior distributions of the frequency of transitions to and from each character state across the tree (visually displayed as a pie chart for each node).

### Phylogenetic PCA morphospace estimation and value permutation

We used phylogenetic principal components analysis Phylogenetic PCA (pPCA) on the dataset of *Silene* floral characters with the ultrametric ITS gene tree to create a floral phenotypic morphospace with the goal of determining whether species cluster by floral morphology. Standard PCA on comparative datasets can mislead inferences (Uyeda et al., 2015); pPCA performs a traditional PCA but corrects for nonindependence among the observations for species using a tree (Revell, 2009). We did not include floral color as a variable in this analysis with the purpose of observing whether morphology is at all associated with floral color. We used all floral character medians as described above, and converted categorical variables to representative integers (e.g., 1, 2, 3 or −1, 0, 1, specified in Supplementary Tables 3, 4). We carried out pPCA analysis using the “phyl.PCA” function in phytools [v1.0–1; (Revell, 2012)] which was used with eigenvalue decomposition of the correlation matrix and phylogenetic signal estimation using Pagel’s λ (Pagel, 1999). We extracted phylogenetically corrected trait correlations by correlating trait values with the pPCs following Berardi et al. (2016) and report correlation coefficients in Supplementary Table 3. Given the phylogenetic signal was low for this dataset, we also performed a standard PCA using the base R function “princomp” for comparison [R v4.1.2; R Core Team (2021)]. To visualize floral morphospace, we plotted pPC1, pPC2, and pPC3 against each other in pairs. We then graphically overlaid floral color as well as ploidy post-analysis to determine whether there was grouping by phenotype. Confidence intervals were calculated for each floral color using “stat_ellipse(type = “norm”)” in ggplot2, which draws a 95% confidence interval from multivariate normal distribution.

We hypothesized that red flower color mean and variance for pPC1 and pPC2 were not different due to chance. To determine whether the mean and variance of floral color group pPC1 and pPC2 scores differed from the null expectation, we used a permutation test. We compared the ratio of variances as well as the differences in mean values between each floral color group in pairwise comparisons for pPC1 and pPC2 separately. For each test, we used 1,000 random permutations of the data with flower color group relabeling, and statistical significance was calculated as the number of times the simulated differences were larger than the empirical differences. Permutations were carried out with R packages coin [v1.4–2, Hothorn et al. (2008)], purrr [0.3.4, Henry and Wickham (2022)], and dplyr [v1.0.8, Wickham et al. (2022)].

To specifically address the robustness of median trait values representing trait ranges for continuous traits (calyx height, calyx width, petal limb length), we randomly selected a value between each species trait minimum and maximum values using a uniform distribution [runif function in the R stats package, v 4.1.2, (R Core Team, 2021)] to generate 1,000 re-sampled datasets. We re-ran the pPCA for each of the 1,000 datasets, and calculated the mean and variance for re-sampled pPC1 and pPC2.

### Phylogenetic distance and phenotypic disparity

We calculated phylogenetic distance on the non-ultrametric ITS tree using the cophenetic.phylo function in R package ape, which computes the pairwise tip distances using branch lengths. We calibrated the ITS tree for the pPCA using the chronopl function in the R package ape [v.5.6–1, Paradis et al. (2004)], assigning an age minimum of 15.14 Myr and maximum of 26.49 Myr to the root of the tree based on Caryophyllaceae dating in Frajman et al. (2009).

We calculated phenotypic disparity for pPC1 and pPC2 for each species pair as the absolute value of the difference [e.g., abs(pPC1.speciesA – pPC1.speciesB) and abs(pPC2.speciesA – pPC2.speciesB)]. We then examined whether species pairwise pPC1 and pPC2 phenotypic disparity was any different when specifically looking at pairs of species of the same and different floral colors (comparisons: red-red, pink-pink, white-white, red-pink, red-white, and pink-white) using nonparametric Kruskal–Wallis tests with the R stats package [v 4.1.2, (R Core Team, 2021)] as well as the R package ARTool for two-way aligned-rank ANOVA (v 0.11.1, Kay et al., 2021).

Since phenotypic disparity as calculated is not specifically corrected for phylogenetic distance, we ran a robust rank-based ANCOVA each for pPC1 and pPC2 disparity as dependent variables, fixed effect sympatric/allopatric and phylogenetic distance as a covariate. We followed the protocol for rank-based ANCOVA in Kloke and McKean (2015) and used the onecovahomog function in R package “npsm” (v 0.5; Kloke and McKean, 2014).

### Species geographic range estimation and analyses

To determine whether species pairs have geographic overlap (sympatry) or not (allopatry), we estimated species ranges and calculated overlap. Specifically, we followed the first three scripts of the gatoRs pipeline, available on Github (Patten et al., n.d.).^1^ Briefly, the R scripts collate species occurrence records from the GBIF, iNaturalist, and iDigBio databases, correct for taxonomic name changes, and clean observations (remove incorrect GPS points, botanical garden observations, etc.) and thin observation points. We then used the sf package [v0.7.6; (Pebesma, 2018)] in R (v3.6.1) to assign the Albers Equal Area projection and the WGS84 CRS to each species observation point. We added a buffer of 10 km^2^ to each species polygon and calculated the area. Last, we calculated the geographic range intersection (overlap) of each species pair as the area of overlap (union of the two polygons) divided by the sum of the non-overlapping areas of both taxa: area of overlap/[(speciesA area – area of overlap) + (speciesB area – area of overlap)] in km^2^ (Phillimore et al., 2008; Li et al., 2018; Hamilton and Wessinger, 2022). If the calculated area of overlap was zero, we considered the species pair to be allopatric, and if the area of overlap was greater than zero, we considered the species pair to be sympatric.

We compared phenotypic disparity of pPC1 and pPC2 between sympatric and allopatric pairs (sympatry and allopatry as categorical variables) using nonparametric Kruskal–Wallis tests with the R stats package [v4.1.2, (R Core Team, 2021)].

### Statistical analysis and graphing

All statistical analyses were performed in R v4.1.2 unless otherwise noted (R Core Team, 2021). R-based plots were generated using ggplot2 [v.3.3.5, Wickham, 2016].

## Results

### Morphological variation in native North American *Silene*

We gathered morphological trait and ploidy data for 47 *Silene* species that are native to North and Central America, most of which are described in the online Flora of North America (Morton, 2022) and some Californian endemics which are described in the Jepson Eflora (2022). We discarded species that have been introduced (e.g., *S. latifolia*, *S. vulgaris,* and *S. coronaria*) to North/Central America. Most native *Silene* in North and Central America belong to the monophyletic section *Physolychnis* in subgenus *Behenantha* (Popp and Oxelman, 2007; Jafari et al., 2020), which spans North, Central, and South America, Asia, and the arctic. The majority of *Physolychnis* are polyploid, but at least two species have both diploid and polyploid accessions, while additional native North/Central American diploid species belong to subgenus *Silene*, various sections (Figure 1; Supplementary Figure 1). Floral colors are white, pink, and red; many species are white/pink polymorphic, which we coded as pink, and some white species are pale yellow, which we coded as white.

We observed extensive among-species variation (i.e., coefficients of variation >30%) for all quantitative traits, even when classifying species into floral color bins (Table 1). Petal limb length had the highest CV (coefficient of variation, 103% across all species). Several North American *Silene* species have very short petals (e.g., *S. campanulata* and *S. antirrhina*, ~2 mm), some of which do not extend much beyond the calyx (e.g., *S. drummondii* and *S. invisa,* ≤2 mm), while others are an order of magnitude larger (e.g., *S. nelsonii* ~ 35 mm). Calyx height and width also exhibit large CV values, both just over 40%. Notably, red-flowering species have consistently smaller CV values in all quantitative traits compared to white- and pink-flowering species.

**Table 1:** Summary statistics of continuous floral and leaf traits by floral color.

	Stem length (cm)				Leaves per leaf node			
	white	pink	red	all	white	pink	red	all
Grand mean (± SD)	241.67 (197.77)	217.92 (159.96)	467.14 (278.57)	258.88 (213.20)	2.21 (0.96)	2.08 (1.84)	2.14 (0.71)	2.10 (1.32)
Grand median	237.5	217.5	550	250	2	2	2	2
CV (%)	81.84	73.41	59.63	82.36	43.63	88.36	33.00	62.76
phylogenetic ANOVA	*F*_(3,43)_ = 5.52, **p = 0.018** Post-hoc: red vs. pink, t = −3.31, df = 25, **p = 0.003**				*F*_(3,43)_ = 0.047, *p* = 0.985			
phylogenetic signal	*--*				*--*			
	**Leaf length (mm)**				**Petal limb length (mm)**			
**white**	**pink**	**red**	**all**	**white**	**pink**	**red**	**all**
Grand mean (± SD)	38.28 (23.46)	44.29 (34.30)	42.79 (23.68)	41.75 (27.91)	5.18 (8.47)	6.94 (6.40)	11.50 (4.09)	6.86 (7.05)
Grand median	38.75	45	45	40	5.75	5.125	10.5	6
CV (%)	61.30	77.44	55.34	66.86	163.38	92.16	35.57	102.72
phylogenetic ANOVA	*F*_(3,43)_ = 0.135, *p* = 0.931				*F*_(3,43)_ = 0.850, *p* = 0.614			
phylogenetic signal	*--*				Blomberg’s K = 0.056 Pagel’s λ = 0.165			
	**Calyx width (mm**)				**Calyx height (mm)**			
**white**	**pink**	**red**	**all**	**white**	**pink**	**red**	**all**
Grand mean (± SD)	5.47 (2.98)	5.13 (1.60)	5.43 (1.17)	5.35 (2.23)	11.75 (5.01)	15.04 (6.92)	19.07 (4.53)	14.23 (6.12)
Grand median	5.75	5	5.5	5.5	12.25	14.25	19	14
CV (%)	54.46	31.25	21.49	41.71	42.64	46.02	23.77	43.01
phylogenetic ANOVA	*F*_(3,43)_ = 0.520, *p* = 0.767				*F*_(3,43)_ = 2.87, *p* = 0.153			
phylogenetic signal	Blomberg’s K = 0.0193 Pagel’s λ = 0.128				Blomberg’s K = **0. 0479** Pagel’s λ = 0.244			

The median, mean, and standard deviation (SD) were calculated from medians of the species’ trait ranges. Coefficient of variation (CV) was calculated as (SD/mean * 100). *N* = white (20), pink (18), red (9), total (47). Phylogenetic ANOVAs were performed on each trait to determine whether each trait varies by floral color with a pairwise *post-hoc t*-test using the Holm method of multiple comparison correction. Phylogenetic signal was calculated for floral traits as Blomberg’s K and Pagel’s λ, and in bold if statistically significant.

Categorical trait values such as inflorescence class, floral tube extension, and reproductive organ exsertion were distributed across floral color classes, and floral color did not significantly explain variation among any of these traits (phylogenetic ANOVA; Table 2). These traits were generally recorded in the FNA as the categories presented here, which likely reflect the simple classification of complex traits that may require more precise observation. For example, reproductive organ exsertion is a nuanced trait in *Silene,* with most species displaying some degree of androecia and gynoecia exsertion above the corolla. Field observations as well as photographs and plate drawings suggest that many pink- and red-flowering species incorporate additional spatial organization in the reproductive organ arrangement, especially the extension and gathering of androecia toward the top of the flower in red species (Hitchcock and Maguire, 1947; Chowdhuri, 1957; Morton, 2022), A. Berardi, *personal observation*).

**Table 2:** Summary of proportions per flower color class for each categorical variable inflorescence class (number of flowers per inflorescence), floral tube extension from the calyx, reproductive organ exertion, and ploidy.

Floral color^*^: (# species)	White (20)	Pink (18)	Red (9)	All (47)
**Inflorescence class (# flowers per inflorescence)**				
One	0.15	0.39	0.11	0.23
Few (1–3 flowers)	0.60	0.33	0.89	0.55
Many (>3 flowers)	0.25	0.28	0.00	0.21
phylogenetic ANOVA	*F*_(3,43)_ = 0.780, *p* = 0.654			
phylogenetic signal	Blomberg’s K = 0.0461 Pagel’s λ = 6.61e^−5^			
**Floral tube extension from calyx**				
above	0.35	0.11	0.33	0.26
equal	0.40	0.72	0.67	0.57
below	0.25	0.17	0.00	0.17
phylogenetic ANOVA	*F*_(3,43)_ = 2.21, *p* = 0.234			
phylogenetic signal	Blomberg’s *K* = 0.0322 Pagel’s λ = 7.42e^−5^			
**Reproductive organ exsertion from corolla**				
above	0.50	0.28	0.67	0.45
above/equal	0.15	0.17	0.33	0.19
equal	0.25	0.39	0.00	0.26
below	0.10	0.17	0.00	0.11
phylogenetic ANOVA	*F*_(3,43)_ = 3.41, *p* = 0.091 Post-hoc: red vs. pink t = −2.60, df = 25, **p = 0.03**			
phylogenetic signal	Blomberg’s *K* = 0.0475 Pagel’s λ = 4.70e^−5^			
**Ploidy**				
diploid	0.10	0.11	0	0.09
diploid/polyploid	0.00	0.11	0	0.04
polyploid	0.90	0.78	1	0.87
phylogenetic ANOVA	*F*_(3,43)_ = 2.07, *p* = 0.249			

Phylogenetic ANOVAs were performed on each trait to determine whether each trait varies by floral color with a pairwise *post-hoc t*-test using the Holm method of multiple comparison correction. Phylogenetic signal was calculated for floral traits as Blomberg’s K and Pagel’s λ, and in bold if statistically significant.

* Floral color Blomberg’s *K* = 0.035 and Pagel’s λ = 4.26e^−5^.

Red-flowering species show smaller amounts of categorical trait variation among species than do white- and pink-flowering species. Unlike pink and white species, most red species have the same inflorescence structure, floral tube extension phenotype, and reproductive organ exsertion (Table 2), with longer reproductive organ exsertion than pink-flowering species (phylogenetic *post-hoc* Tukey’s test, *t* = −2.60, df = 25, *p* = 0.03). Red-flowering species have longer stems than pink-flowering species (phylogenetic *post-hoc* Tukey’s test, *t* = −3.30, df = 25, *p* = 0.003) and produce larger flowers (taller calyces and longer petal limbs) than many (but not all) pink or white species (Table 1). Only a few species in North and Central America are diploid or of variable diploidy/polyploidy, but none of these are red-flowering species.

### Floral trait correlations

**Figure 2:**
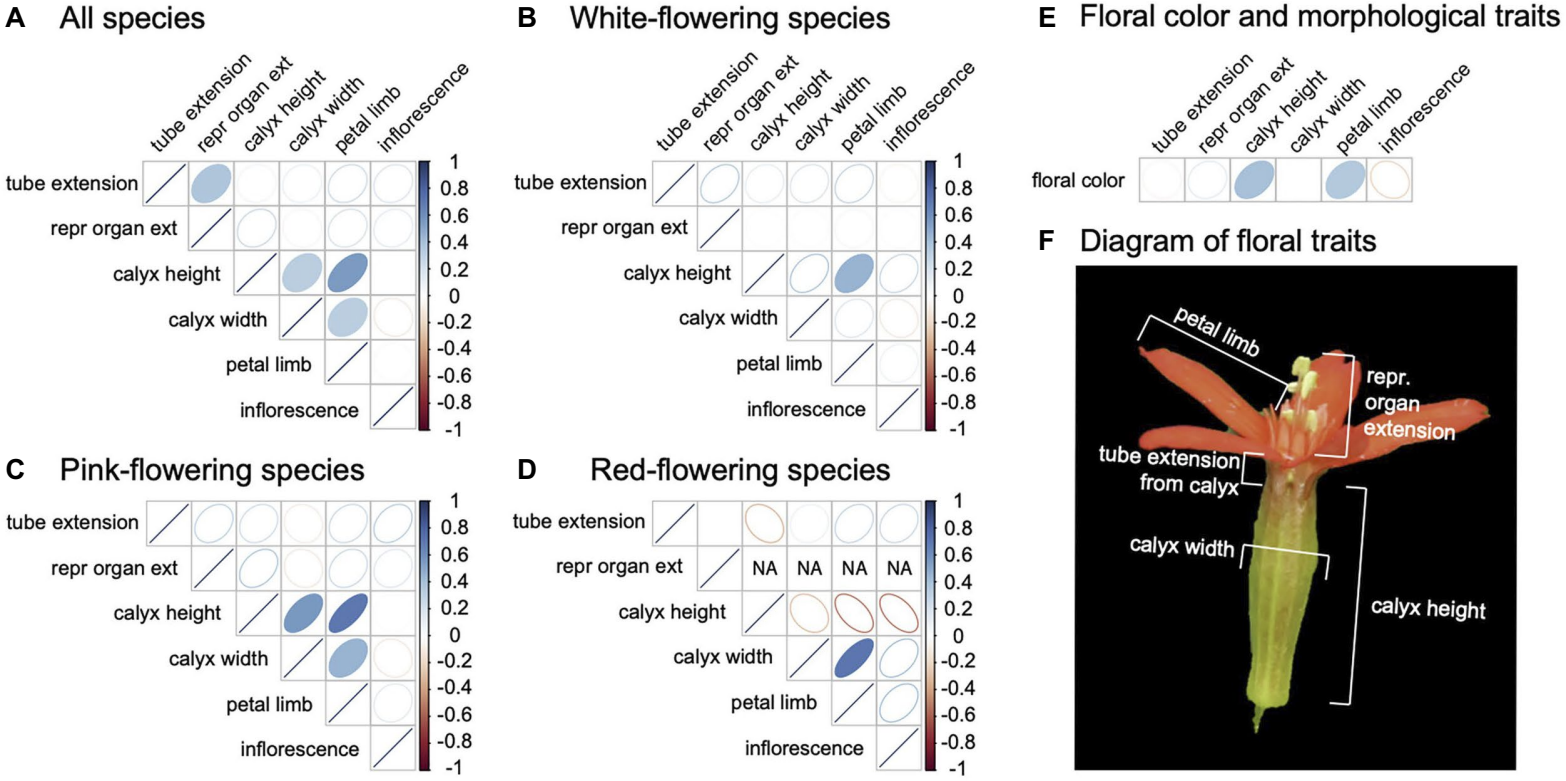
Floral morphological trait correlations in the total species dataset **(A)**, and within each floral color group [white **(B)**, pink **(C)**, red **(D)**]. **(E)** Floral color is correlated with all traits in the total species dataset. Spearman rank correlations are depicted as ellipses, with the strength of Spearman’s rho (r) indicated by color and the direction of the ellipse indicating a positive or negative value. Statistically significant correlations (*p* ≤ 0.05) are shown as ellipses with filled color. Cells with “NA” indicate that all phenotypic values were uniform for the trait, thus a correlation could not be calculated. **(F)** Traits included in this analysis, apart from inflorescence (see methods), are depicted on an *S. regia* flower. Samples sizes for all species (*n* = 47), white-flowering species (*n* = 20), pink-flowering species (*n* = 18), red-flowering species (*n* = 9).

We quantified morphological trait correlations to determine if floral evolution across the clade converged on distinct and divergent floral phenotypes or “syndromes.” We observed floral trait correlations without phylogenetic correction to allow us a greater sample size (pPCA analysis using the correlation approach suggested that phylogenetic signal for the dataset is low, thus phylogenetic and standard correlation values are nearly identical, Supplementary Table 3). We considered trait correlations across all species, and then across species within a flower color group (Figure 2**)**. In general, there were few significant correlations, suggesting independent evolution of floral traits across the phylogeny with weak or absent grouping of particular floral traits into syndromes (Figure 2A). Across all species, white-flowering species, and pink-flowering species, we found some weak positive correlations between aspects of flower size. White-flowering species showed a positive association between calyx height and petal limb length, and pink-flowering species showed a positive association between calyx height, calyx width, and petal limb length. Although we did find that red flowers have a positive correlation between calyx width and petal limb length, we found a negative correlation between calyx height and other floral traits. While these correlations are not significant, likely due to low statistical power with small sample size, they are markedly different than the correlations seen in other flower color groups. Tube extension from the calyx was the same value (tube above calyx) in all red species, so a correlation could not be calculated.

### ITS tree and the origin of red flower color

We used publicly available internal transcribed spacer (ITS) sequences from 37 species to estimate a tree and to observe the distribution of floral color and ploidy along the tree. While only a cursory view into the evolution of these traits (a single-locus tree does not always match the species tree), ITS has been used to resolve the major phylogenetic grouping in North American *Silene* (Burleigh and Holtsford, 2003; Popp and Oxelman, 2007; Mesler et al., 2020). The ITS tree (Figure 1B; Supplementary Figure 1) reflects previously demonstrated phylogenetic grouping for North and Central American *Silene*, including monophyletic groupings of *Physolychnis* s.l. and the *S. menziesii* group (Popp and Oxelman, 2007). Similar to Popp and Oxelman (2007) we also observed that the red-flowering species *S. laciniata* is not monophyletic, with different subspecies placed in different locations within *Physolychnis*. We therefore treated the subspecies as separate entries for phenotypic analysis. We mapped floral color and ploidy traits to the ITS tree (Figure 1B) and noted that all red-flowering species are indeed polyploid and members of section *Physolychnis*. Red-flowering species are distributed across the *Physolychnis* group in the tree, suggesting a single red-flowering ancestor is unlikely (Figure 1B).

Next, we performed an ancestral state reconstruction to determine whether red flower color likely arose from a single or multiple evolutionary events within section *Physolychnis,* as well as to determine whether red flower color is a more recent phenotype than pink or white. The best-fitting ML model for discrete floral color evolution was the “equal rates” model, also known as the Mk1 model, as it had the lowest AIC score. The second best-fitting model was the stepwise model with reversible stepwise transitions from white to pink to red floral color, but not white to red or red to white (transitions must proceed through pink; see Supplementary Table 1 for descriptions of all models). Since the next best-fitting model was less than two AIC units higher, we ran both models (Supplementary Figures 1A,B). In the ER model, the most likely state for transitions to red is from pink, and the most frequent state change is from pink to white (loss of color; Supplementary Table 2). More importantly, we observe in both models that most transitions to red have likely occurred recently since most ancestral nodes do not show strong support for red ancestors. Given the low support for red flower color arising early or before the *Physolychnis* section was established, we infer that red flower color is indeed a recently evolved trait. Furthermore, we can infer multiple independent gains of red flower color, most often deriving from pink but also white ancestors (e.g., *S. subciliata* and *S. laciniata ssp. californica* from white ancestors). Multiple independent transitions suggest that red floral color is a convergent phenotype, with the single exception of *S. rotundifolia* and *S. regia* which form a sister pair. Overall, the evolution of red flower color appears to be repeated and complex.

### Floral morphospace

We used a phylogenetic principal components analysis (pPCA) on floral traits to construct a floral morphospace for North American *Silene* with a modified ITS gene tree (modified from the tree represented in Figure 1B, where we only used a single ITS copy per lineage). Phylogenetic signal was low for the pPCA analysis (Pagel’s λ = 7.56e-05) and thus had similar results to a standard PCA calculated from the correlation matrix (Supplementary Figure 3). Additionally, each trait displayed low phylogenetic signal (Tables 1, 2).

The pPCA partitioned the variance of the floral morphological data into principal components (pPCs) that tended to cluster species by relative floral size and reproductive organ extension. Collectively, the first three pPCs explained 70% of the variance (Supplementary Table 4). We examined the loadings from the pPCA for broad patterns of correlations among the floral traits and the pPCs. pPC1 (explaining 29.9% of the total variance) was strongly and negatively associated with floral tube extension, reproductive organ exsertion, calyx height, and petal limb length, indicating that high values of pPC1 represent smaller with low to no floral tube or reproductive organ extension, and negative values of pPC1 represent larger and extended flowers as well as larger overall floral displays. pPC2 (explaining 23% of the variance) is strongly associated with calyx width and negatively correlated with inflorescence class; high values of pPC2 represent flowers with wider calyces and fewer flowers per inflorescence. pPC3 (explaining 17% of the variance) is moderately associated with floral tube extension and negatively correlated with calyx height.

**Figure 3:**
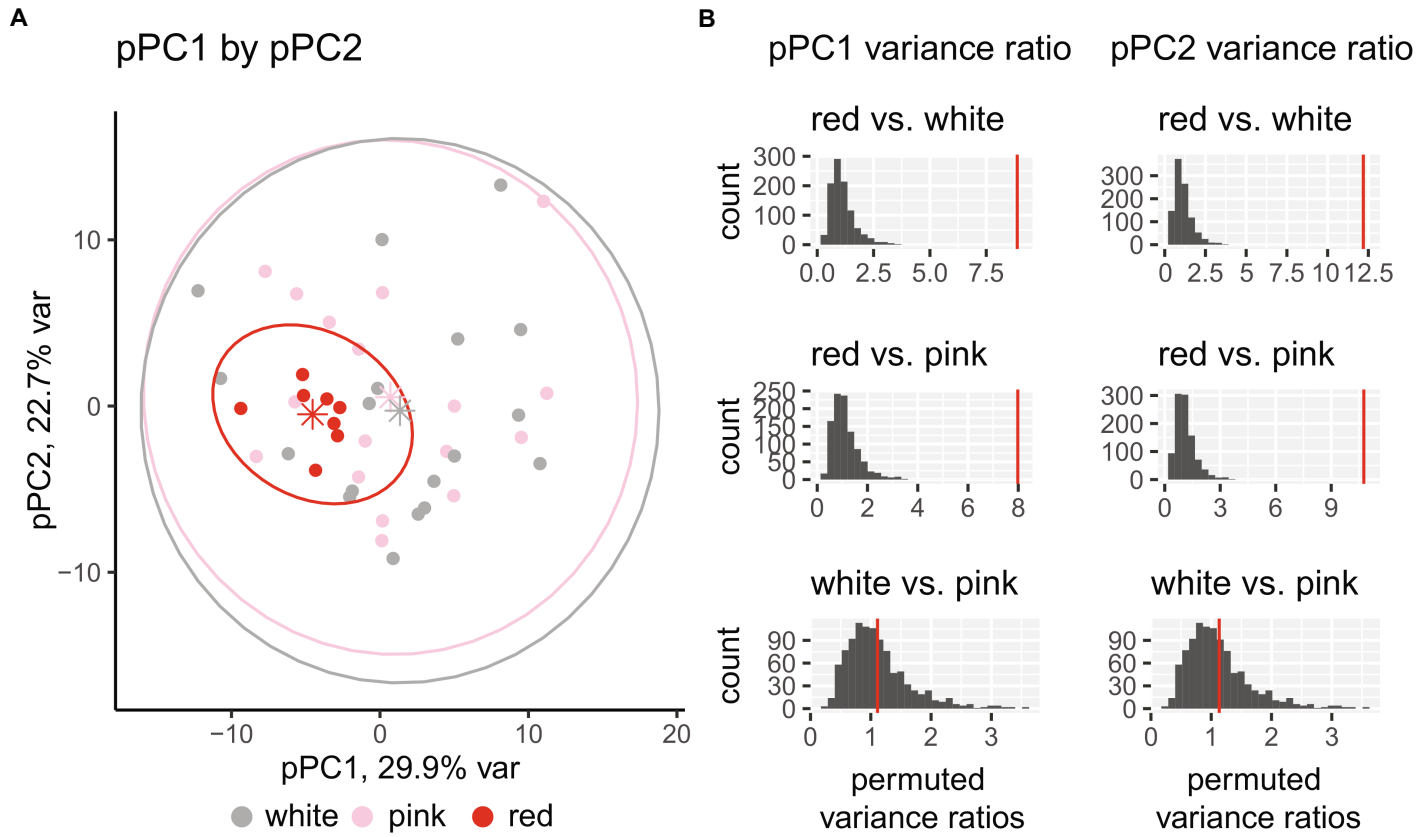
**(A)** Phylogenetic PCA (pPCA) of floral morphological trait variation. Each species with a corresponding ITS sequence (*N* = 43) is represented by a single dot, with the color indicating the floral color. Floral color points are bounded by ellipses representing 95% confidence intervals. Mean pPC values for each floral color group are represented as stars. **(B)** Ratio of variances between each flower color for both pPC1 and pPC2 with empirical values as red vertical lines and distribution of 1,000 randomized permutations shown in gray bars. Permutation tests demonstrate that red-flowering species have much lower morphological variance compared to both pink- and white-flowering species (*p* < 0.05, one-sided permutation test of the ratio of variances). The difference in variance between white- and pink-flowering species is not statistically significant.

When the floral morphological pPC observations are plotted with floral color overlaid on each species (Figure 3A), red-flowering species cluster closely together inside the total morphospace. This pattern holds consistent for comparing pPC1 with pPC3 and pPC2 with pPC3 (Supplementary Figure 3). This consistent and close clustering of red-flowering species in the pPC morphospace suggests red flowers represent a subsampling (or sorting) from standing phenotypic variation in *Silene* rather than *de novo* adaptations into a new, unoccupied, phenotypic space. Red-flowering species reside in morphospace described by long and narrow calyces, extended floral tubes and reproductive organs, and long petals. The phenotypic mean is most distinct along pPC1, where red-flowering species have lower values than do white- and pink-flowering species (including a significant difference in mean pPC1, Supplementary Figure 4). Conversely, pink- and white-flowering species overlap across the entire morphospace, representing all aspects of floral diversity of North American *Silene* regardless of floral color. The variance of red-flowering species as a group is much smaller than either white- or pink-flowering species, as depicted by the size of the 95% confidence interval ovals in Figure 3A. To determine whether the reduced variance was significantly less, we randomly permutated species pairwise ratios of variance while re-assigning floral color (Figure 3B). Red-flowering species have much lower morphological variance when compared to pink- and white-flowering species (*p* < 0.05, one-sided permutation test of the ratio of variances) while there was no difference between white- and pink-flowering species.

We recognize that using trait medians as reported from the literature for each species may bias our conclusions about divergence in trait values and trait variances. To test this bias we randomly re-sampled continuous trait values for each species from a uniform distribution that fell between the reported minimum and maximum values from the literature. We generate 1,000 re-sampled datasets and re-ran the pPCA. Consistent with our analyses on the median values, the variance of red-flowering species is much lower than that of white or pink-flowering species across all three major axes of variation, and pPC1 (and not pPC2) shows differences in the means among floral color classes (Supplementary Figure 5). pPC morphospaces continued to show strong phenotypic overlap in pink and white species, with red species occupying a smaller space nested inside the pink and white species’ phenotypes (Supplementary Figure 6).

We explored whether ploidy levels informed interpretation of the floral morphospace. We found that pPC1 separated the majority of polyploids from the diploid or diploid/polyploid polymorphic species (Supplementary Figure 7). Polyploidy is associated with negative values of pPC1, indicating larger and showier flowers than the related diploid lineages.

### Effects of geographic overlap, sympatry, and allopatry on floral morphology

**Figure 4:**
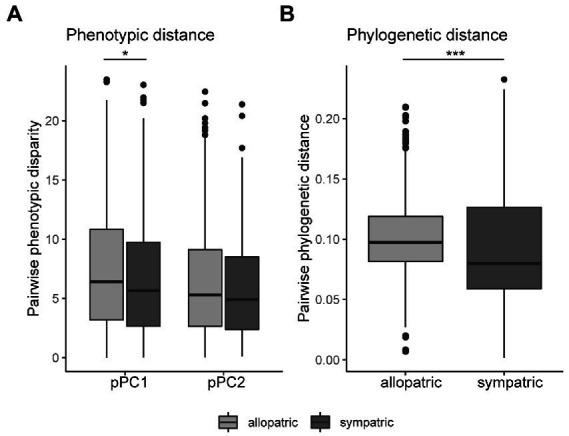
Phenotypic and phylogenetic distance between sympatric and allopatric species pairs. **(A)** Species with entirely allopatric range distributions show greater phenotypic disparity in floral pPC1 (representing size), but not floral pPC2 (width/inflorescence), compared to species pairs that share some or geographic range (sympatry; pPC1: Kruskal–Wallis *X*^2^ = 4.95, df = 1, * *p* = 0.026; pPC2: Kruskal–Wallis *X*^2^ = 1.21, df = 1, *p* = 0.27). **(B)** Phylogenetic distance is greater between species pairs in allopatry than in sympatry (Kruskal−Wallis *X*^2^ = 18.369, df = 1, ***p < 0.001).

Disruptive selection to reduce competition (i.e., for pollinators) or reproductive interference can drive divergence between geographically overlapping species. Thus, selection for floral divergence may be stronger between sympatric species than species with entirely allopatric ranges. For this reason, we may expect to see greater signatures of divergence in floral morphology between species that share geographic range overlap than species that are purely allopatric. We calculated phenotypic disparity for pPC1 and pPC2 for each species pair and classified the species pair as sympatric if their geographic ranges overlapped at all (>0 km). While floral pPC1 disparity was significantly different between allopatric and sympatric pairwise comparisons (Kruskal–Wallis *X*^2^ = 4.95, df = 1, *p* = 0.026), the median pPC1 phenotypic disparity in sympatry was actually slightly lower than in allopatry (Figure 4A, sympatric median at 5.67, allopatric at 6.40). The same general pattern was detected in pPC2 disparity, although not statistically significant (Figure 4A, sympatric median at 4.90 and allopatric at 5.30).

Although phylogenetic signal for floral traits was weak, we repeated this analysis using phylogenetic distance as a covariate (since disparity is no longer specifically corrected for phylogeny) and found qualitatively similar results with no more divergence in sympatric than allopatric pairs (rank-based robust ANCOVA: pPC1 disparity *F* = 4.15, *p* = 0.042 allopatric > sympatric; pPC2 disparity *F* = 1.032, *p* = 0.310). We did find that phylogenetic distance was greater in allopatric species pairs than in sympatric pairs (Figure 4B).

Furthermore, there was no association between the degree of sympatry in pPC1 or pPC2 (i.e., the area of overlap in km^2^ and range overlap; Spearman’s *r* = −0.062 to-0.044 for pPC1 and pPC2, *p* = 1).

We additionally considered floral color as a second categorical variable in phenotypic disparity. Taking a nonparametric two-way ANOVA approach (using aligned rank transformed data), we determined whether phenotypic disparity differed by floral color comparison (e.g., red–red, white–white, pink–pink, red–pink, red–white, white–pink) in pPC1 and pPC2, and whether resulting phenotypic disparity differed between sympatric or allopatric species pairs. Phenotypic disparity in pairwise species comparisons was only explained by floral color comparison (pPC1 disparity: *F*_(5,808)_ = 2.42, *p* = 0.034; pPC2 disparity: F_(5,808)_ = 3.88, *p* = 0.0018), and not by sympatry/allopatry. The likely driver of statistical significance of floral color comparison predicting phenotypic disparity is the red-red comparison, where there is very low phenotypic disparity across red-flowering species (Supplementary Figure 8). This result is in line with the close phenotypic clustering of red species observed in pPC1 and pPC2 morphospace.

## Discussion

Observing trait correlations across independently evolving lineages can indicate convergent evolution with particular trait combinations evolving repeatedly in response to a similar selection pressure. It is this repeated selection by particular pollinators that has led to the evolution of correlated floral traits into well-characterized pollination syndromes across diverse angiosperm clades. However, such patterns of convergent evolution can fail to evolve or can be difficult to observe for several non-mutually exclusive reasons: (1) pollinator-mediated selection is not strong, (2) a taxonomic group has not had enough evolutionary time to diverge, (3) the species have many effective pollinators, thus favoring a more generalized floral form, and (4) the species are more constrained by similar evolutionary history than they are influenced by divergent pollinator selection. In the North American *Silene*, we find limited evidence of floral divergence that would indicate the evolution of strict pollination syndromes, and yet we do find some evidence of convergence in floral form in red-flowering species. Our findings reveal extensive phenotypic variation across the clade with red-flowering species occupying a specific subset of that variation.

### Floral phenotypic variation is large, but red species exist in a subset of morphospace

Phenotypic variance in North American *Silene* is substantial in both floral and vegetative traits. This diversity likely reflects extensive genetic variation, multiple polyploidization events, specialization to numerous habitats, and possibly adaptation to pollinators. Due to the lack of detailed pollination studies for most North American *Silene*, we cannot easily infer pollination syndromes using species with known pollinators as indicators (Wilson et al., 2004; Jürgens, 2006; Ollerton et al., 2009; Bröderbauer et al., 2013; Chartier et al., 2014; Serrano-Serrano et al., 2017). Instead, we looked for correlated patterns of trait evolution that would be consistent with convergent responses to selection. We find extensive phenotypic variation in floral morphology, but no clustering of variation into separated morphogroups (Figure 3A; Supplementary Figures 2, 3). Low levels of phenotypic disparity among species with different pollinators can certainly occur, especially in groups where repeated pollinator shifts have occurred, e.g., in *Ruellia* where insect pollination repeatedly arose from hummingbird-pollinated ancestors and retained some ancestral morphological similarity (Tripp and Manos, 2008).

Delineating North and Central American *Silene* species in morphospace by floral color reveals that red-flowering species converge upon a smaller section of the morphospace with much less phenotypic diversity than white- or pink-flowering species. Red flower color likely evolved in *Silene* section *Physolychnis* more than once, and these species have converged upon a narrow section of the floral phenotypic variance characterized by floral tube extension from the calyx, reproductive organ extension, and long calyces (and floral tubes) of moderate width. While red species have low phenotypic disparity from each other, it is important to note that they are still entirely nested within the broader *Silene* morphospace. Therefore, these “red” trait values and even trait combinations are not unique. White- and pink-flowering species *S. occidentalis*, *S. parishii*, *S. douglasii*, *S. caroliniana*, *S. bernardina, S. scouleri, S. stellata,* and *S. scaposa* floral phenotypes fall within the 95% confidence interval of red-flowering species, and not all are closely related to red species (Figure 3A; Supplementary Figure 3A). Given the low morphological differentiation among the species, floral color may be one of the key differentiators for pollinator discrimination and preference. Floral color is a powerful signal that can be one of the first traits that inform a pollinator’s choice (Hoballah et al., 2007; Hopkins and Rausher, 2011; Yuan et al., 2013; Kellenberger et al., 2019), and further research is warranted in this system.

### Sympatry and allopatry are not associated with major floral morphological divergence

Related and sister species are generally predicted to have greater phenotypic divergence in sympatry than in allopatry due to selection to reduce deleterious hybridization (Dobzhansky, 1940; Mayr, 1942) and decrease competition for a shared resource (Brown and Wilson, 1956). This divergence is often observed in key floral reproductive traits, such as in floral morphology and color, and especially in response to competition for pollinators (Grossenbacher and Whittall, 2011; Eaton et al., 2012; Hopkins and Rausher, 2012; Briscoe Runquist and Moeller, 2014; Grossenbacher and Stanton, 2014). For this reason, we might expect to see that geographic context masks a pattern of strong phenotypic divergence in floral traits such that only sympatric species show the pattern of divergence and not allopatric pairs. We did not observe this pattern in North American *Silene* floral phenotypes. When comparing floral phenotypic disparity (pPC1 and pPC2), there was either very little or no difference in the extent of floral divergence between sympatric or allopatric species pairs. In fact, sympatric species pairs were often more (albeit marginally) phenotypically similar than allopatric species (Figure 4). Comparisons between floral colors in sympatry or allopatry were also not significantly different. These findings might suggest that even in geographic overlap, shared pollinator resources and the threat of costly hybridization have not driven extensive pollinator-mediated divergence in floral form.

Our results are surprisingly contrary to our expectations, but there are several possible explanations. First, our measures of phenotypes are based on species medians. This summary may not be sensitive to phenotypic divergence between sister taxa at more local or population-based levels [e.g., *S. virginica* and *S. caroliniana* (Kruckeberg, 1964; Mitchell and Uttal, 1969)]. It is possible that phenotypic divergence is still greatest in areas of strict sympatry. This calls for more specific measurements of species pairs of interest at a more geographically fine scale. Second, there may be other traits, besides floral morphology, that cause reproductive isolation or relieve competition. Studies into flowering time, pollen-pistil interactions, selfing rates, and polyploid parental lineage inheritance may reveal differences that prevent reproduction between species.

### Study limitations and future directions

While this study provides an initial look into morphological floral evolution in North American *Silene,* there are some important limitations. First, our phylogenetic inference is based on a single-locus ITS tree rather than a true species tree. Since our ability to infer intrageneric-level processes is limited to the evolutionary history of ITS, so too are our ancestral state reconstructions. Despite this, it is evident that red flower color arose more than once in *Physolychnis*, and likely from both pink and white ancestors, representing both gains and shifts in floral color. We can conclude that most red-flowering species have converged (rather than retained strictly from shared ancestry) upon a similar floral phenotype more than white- or pink-flowering species. Further phylogenetic efforts will lend greater insight into the speciation, polyploidization, and diversification history of *Silene*, the identification of parental lineages, and character evolution.

Second, extending our phenotypic measurements to more quantitative rather than categorical values can increase our resolution and may provide more clarity in evolutionary patterns. Additionally, adding phenotypes such as floral scent and nectar reward will expand our understanding for both the attraction and reward traits that are likely important for pollinators (Jürgens, 2004; Smith et al., 2008; Reynolds et al., 2009). Increasing the detail and breadth of floral phenotypic distributions as well as including more population-level data across species ranges may capture subtle but important species interactions.

Third, the paucity of pollination data limits the ability to predict the major or most effective pollinator for most North and Central American *Silene* species. Assigning species to floral pollination syndrome through floral morphology, color, etc., to predict the likely effective pollinator is often done, although heavily criticized (Van Der Niet, 2021). One of the cautions of using a predictive approach is the potential false inferences from unrecognized pollination systems (Van Der Niet, 2021). For example, we might predict that all large, white flowering *Silene* are pollinated by nighttime moths given information from *S. stellata* (Reynolds et al., 2009), yet ongoing research in California suggests that bee-flies are the main pollinators of some phenotypically similar species (M. Mesler, personal communication). Likewise, we would like to confidently assign all red-flowering species to hummingbird pollinators, but only anecdotal reports exist for red species (except for *S. virginica*), and some observations suggest that butterfly visitation is frequent. Finally, determining which *Silene* may be entirely or mostly selfing will illustrate the important and repeated self-pollination syndrome that can arise to avoid pollinator competition (Buide et al., 2015).

## Conclusion

We found that the floral morphology in the mostly polyploid section of *Silene* in North America is highly variable. While we found floral variation among species, the distribution of floral variation and lack of grouping into distinct floral morphologies does not support strict adherence to pollination syndromes by morphology alone. When floral color is taken into consideration, red-flowering species appear to have converged on a specific subset of the floral variation without having diverged from the standing variation in the genus. The notable morphological convergence of red-flowering *Silene* species, together with the geographic restriction of red flowers and hummingbirds to North America suggests an association between pollinator and floral traits. Although further research is required, this pattern is consistent with convergence of red-flowering species in response to hummingbird mediated pollinator selection.

## Data availability statement

Publicly available datasets were analyzed in this study. This data and accessions can be found at: https://doi.org/10.5061/dryad.wstqjq2pf and in the article/Supplementary material.

## Author contributions

AEB designed the research and analyzed the data. ACB and AEB collected the data. AEB and RH drafted the manuscript. All authors contributed to the article and approved the submitted version.

## Funding

AEB was supported by the Harvard University Herbaria Research Fellowship. ACB was supported by the E3 REU program at Harvard University (NSF REU-7484170) and the Harvard University GSAS Summer Research Opportunities at Harvard (SROH).

## Conflict of interest

The authors declare that the research was conducted in the absence of any commercial or financial relationships that could be construed as a potential conflict of interest.

## Publisher’s note

All claims expressed in this article are solely those of the authors and do not necessarily represent those of their affiliated organizations, or those of the publisher, the editors and the reviewers. Any product that may be evaluated in this article, or claim that may be made by its manufacturer, is not guaranteed or endorsed by the publisher.

## Footnotes

^1^ https://github.com/soltislab/BotanyENMWorkshops
