# Supplementary material for: Convergence without divergence in North American red-flowering Silene
Source: Front Plant Sci. 2022 Sep 6;13:945806. doi: 10.3389/fpls.2022.945806 (PMC9485837; doi:10.3389/fpls.2022.945806)
Supplement: Supplementary file 1 [file Data_Sheet_1.PDF]

Supplementary Material

Supplementary Figure 1

Ancestral state reconstruction of floral color via stochastic character mapping on an ultrametric ITS gene tree, with single taxon representatives. We sampled unambiguous character histories from the posterior distribution, with 1000 stochastic maps generated. Ancestral nodes are represented as pies and denote posterior probabilities of each floral color. The two best scoring models are shown in **(A)** Equal rates model (Mk1), and **(B)** Stepwise reversible model. Subgenera (subg.) and sections are listed according to Jafari *et al*. 2020. A = subg. *Silene* section *Siphonomorpha* (*S. acaulis, S. nutans*); B = subg. *Silene* section *Auriculatae* (*S. repens*); C = subg. *Silene* section *Anotites*; D = subg. *Behenantha*, incertae sedis; E = subg. *Behenantha* section *Physolychnis*; F = subg. *Silene* section *Sclerophyllae* (*S. antirrhina*).

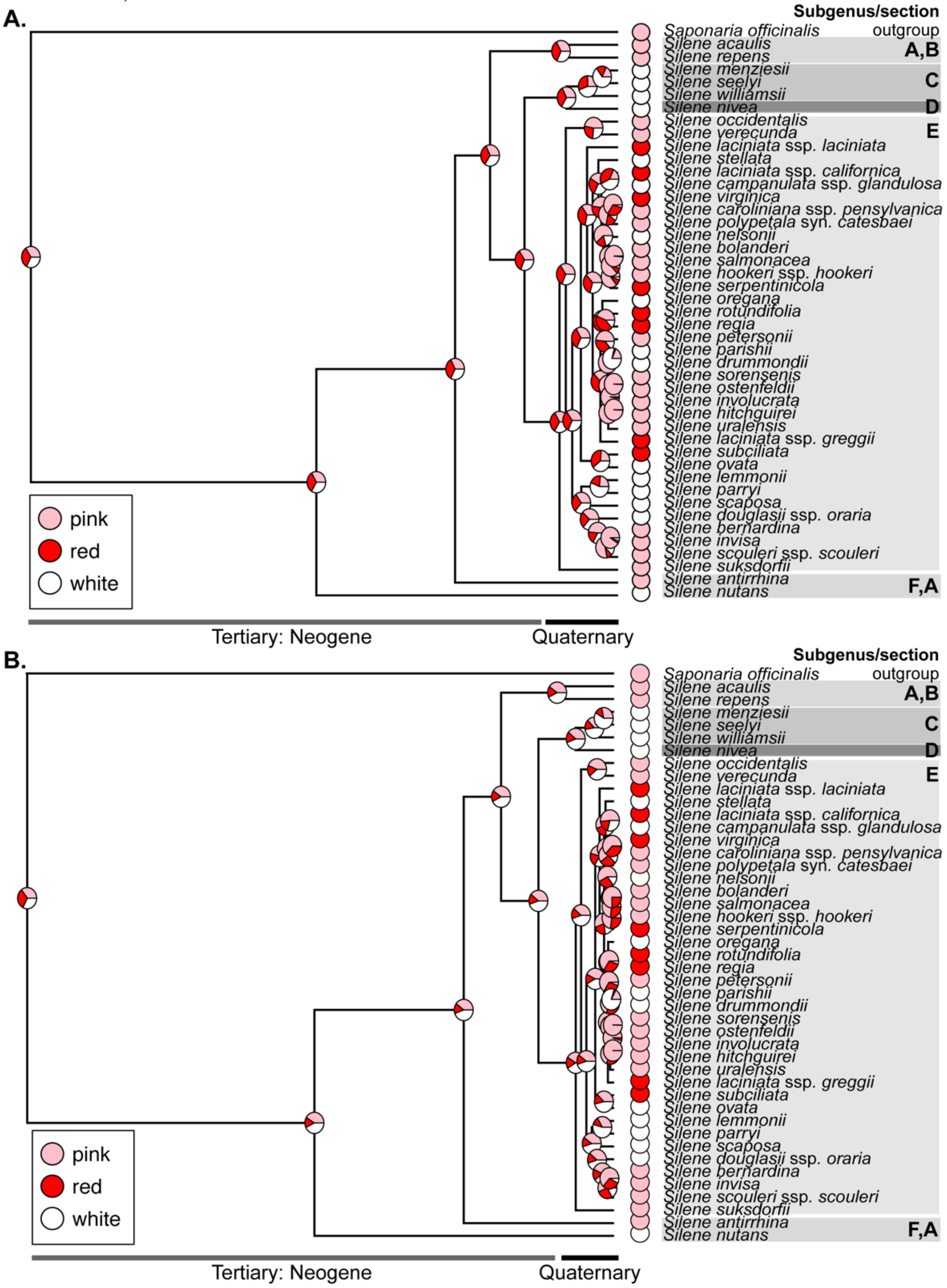

Supplementary Figure 2
PC1 and PC2 scores from the standard PCA on floral morphological traits (n = 47).
(A) Each species is represented by a single dot, with the color representing the floral color (red = red, pink = pink, gray = white). Floral color points are bounded by ellipses representing 95% confidence intervals. PC1 explains 29.4% of the variance and PC2 explains 22.2% of the variance.
(B) Biplot of PC1 and PC2 depicting the PC loadings for the analysis.

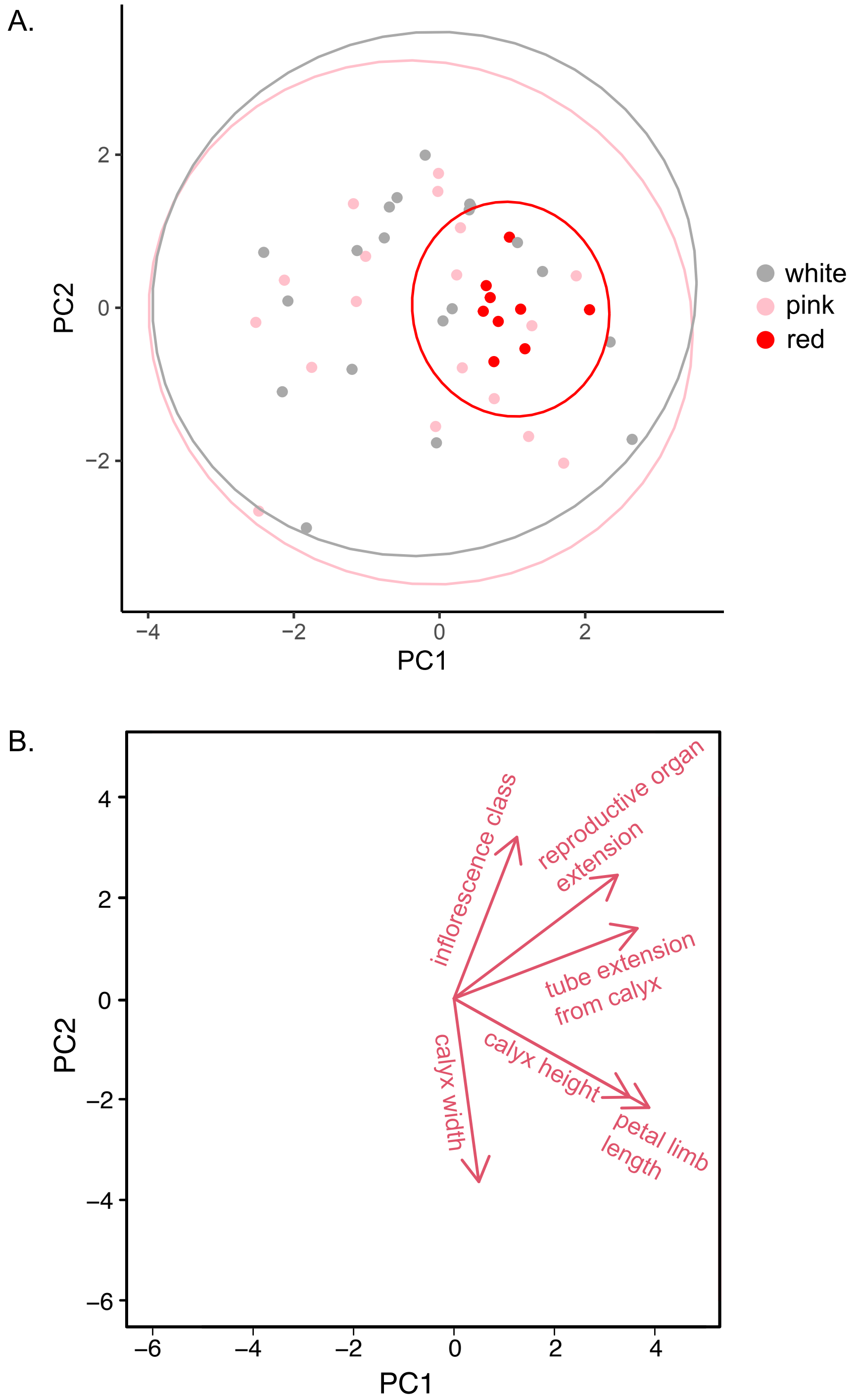

Supplementary Figure 3

(A) Biplot of pPC1 and pPC2 depicting the pPC loadings for the analysis (left), together with species names and locations on the plot (right).

(B) pPC1 vs. pPC3 (left), and pPC2 vs. pPC3 (right) with 95% confidence intervals drawn as ellipses by floral color. Each species is represented by a single dot, with the color of the dot representing the floral color (red = red, pink = pink, gray = white).

A.

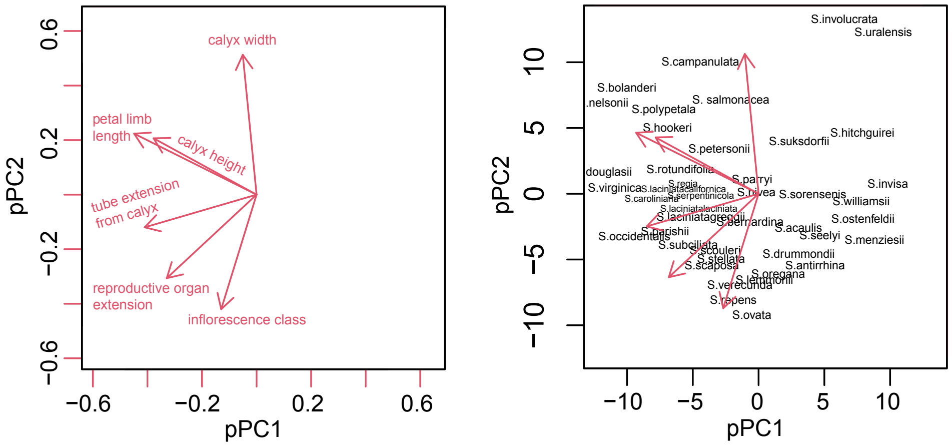

B.

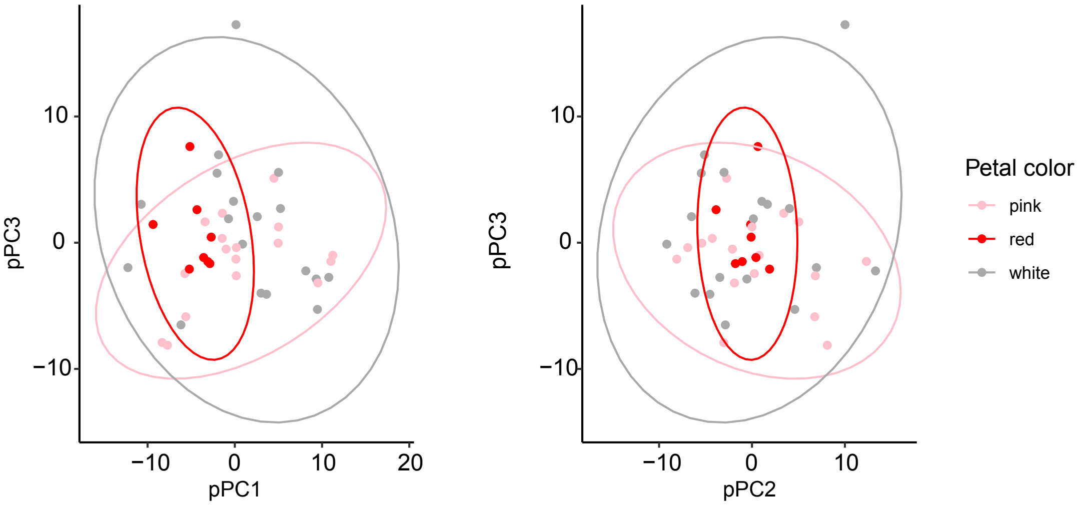

Supplementary Figure 4

Ratio of means between each flower color for both pPC1 and pPC2 with empirical values as red vertical lines and distribution of 1000 randomized permutations shown in gray bars. Permutation tests demonstrate that red-flowering species have a greater mean pPC1 difference than pink- or white-flowering species ($p < 0.05$, one-sided permutation test of the difference between means). No differences in means were detected between pink- and white-flowering species for pPC1, and no significant differences in means were detected for any comparison for pPC2.

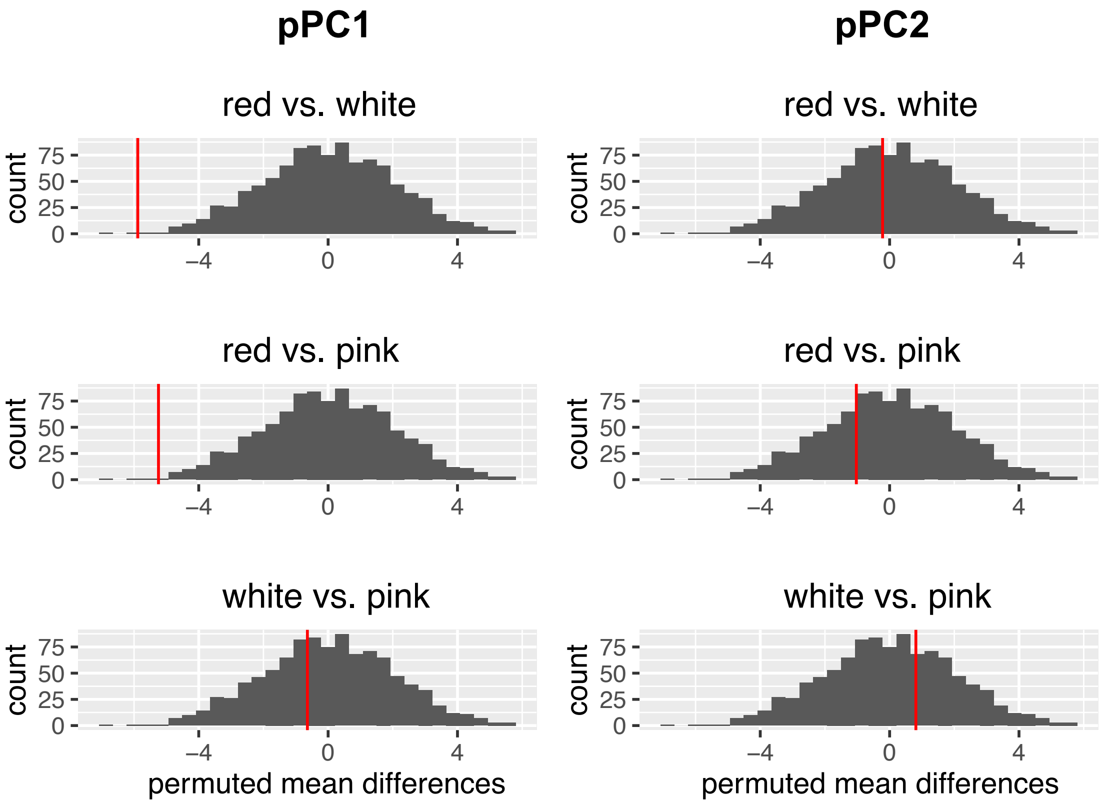

Supplementary Figure 5

To determine whether selecting the median values of species floral trait ranges bias the conclusion that red-flowering species have much less phenotypic variance than do pink- or white-flowering species in phylogenetic morphospace, a value was randomly selected from within the bounds of each species' trait range (between the minimum and maximum values for each trait and species, for continuous traits only) from a uniform distribution and re-calculated the phylogenetic PCA (pPCA) 1000 times.

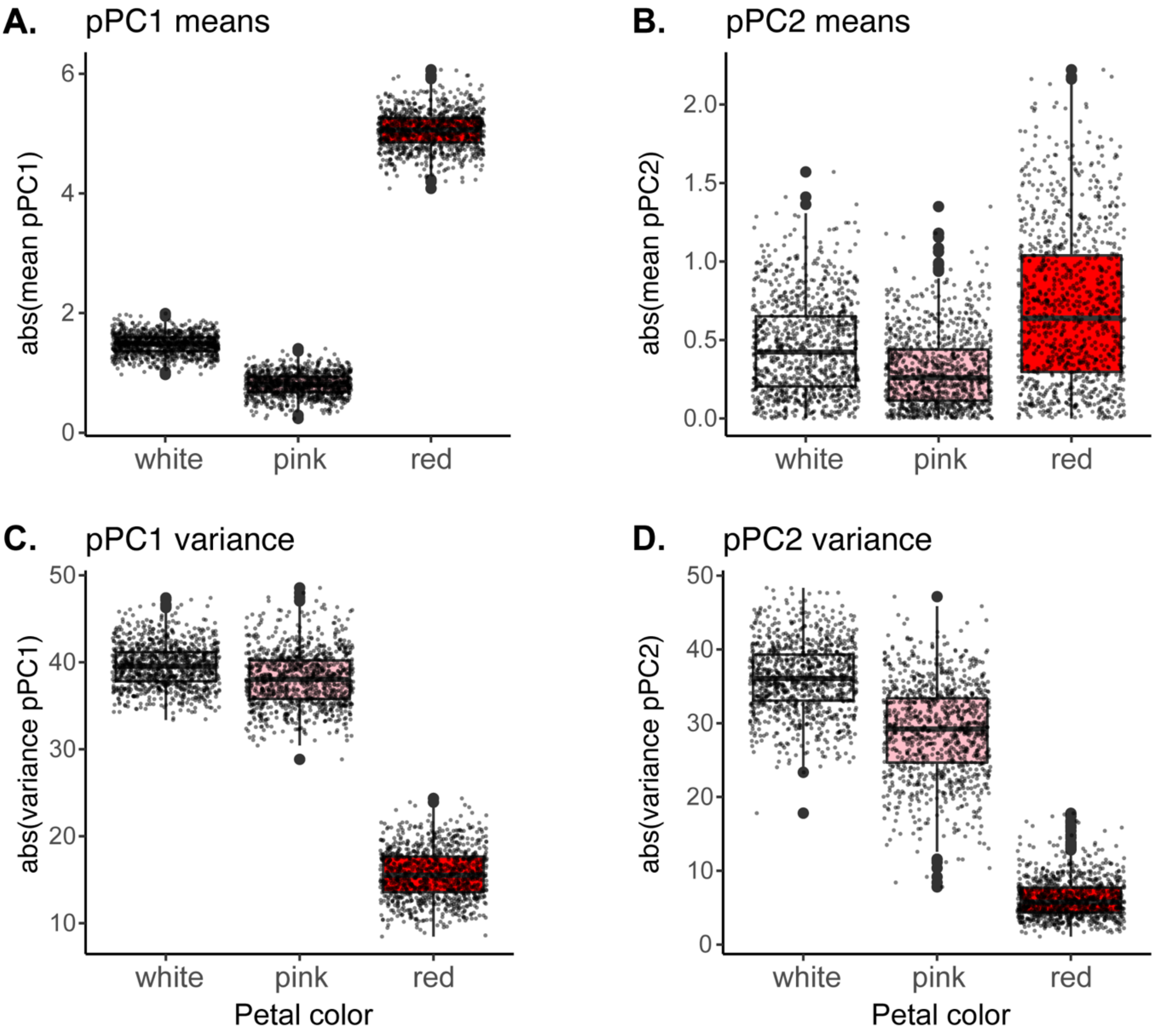

Supplementary Figure 6

To determine whether selecting the median values of species floral trait ranges bias the conclusion that red-flowering species have much less phenotypic variance than do pink- or white-flowering species in phylogenetic morphospace, we randomly selected a value within the bounds of each species' trait range (between the minimum and maximum values for each trait and species) from a uniform distribution and re-calculated the phylogenetic PCA (pPCA) 1000 times. Displayed below are plots of the first 100 of the re-sampled datasets, with each species represented by a dot, and floral color indicated by the color of the dot (red = red, pink = pink, gray = white floral color). Floral color points are bounded by ellipses representing 95% confidence intervals.

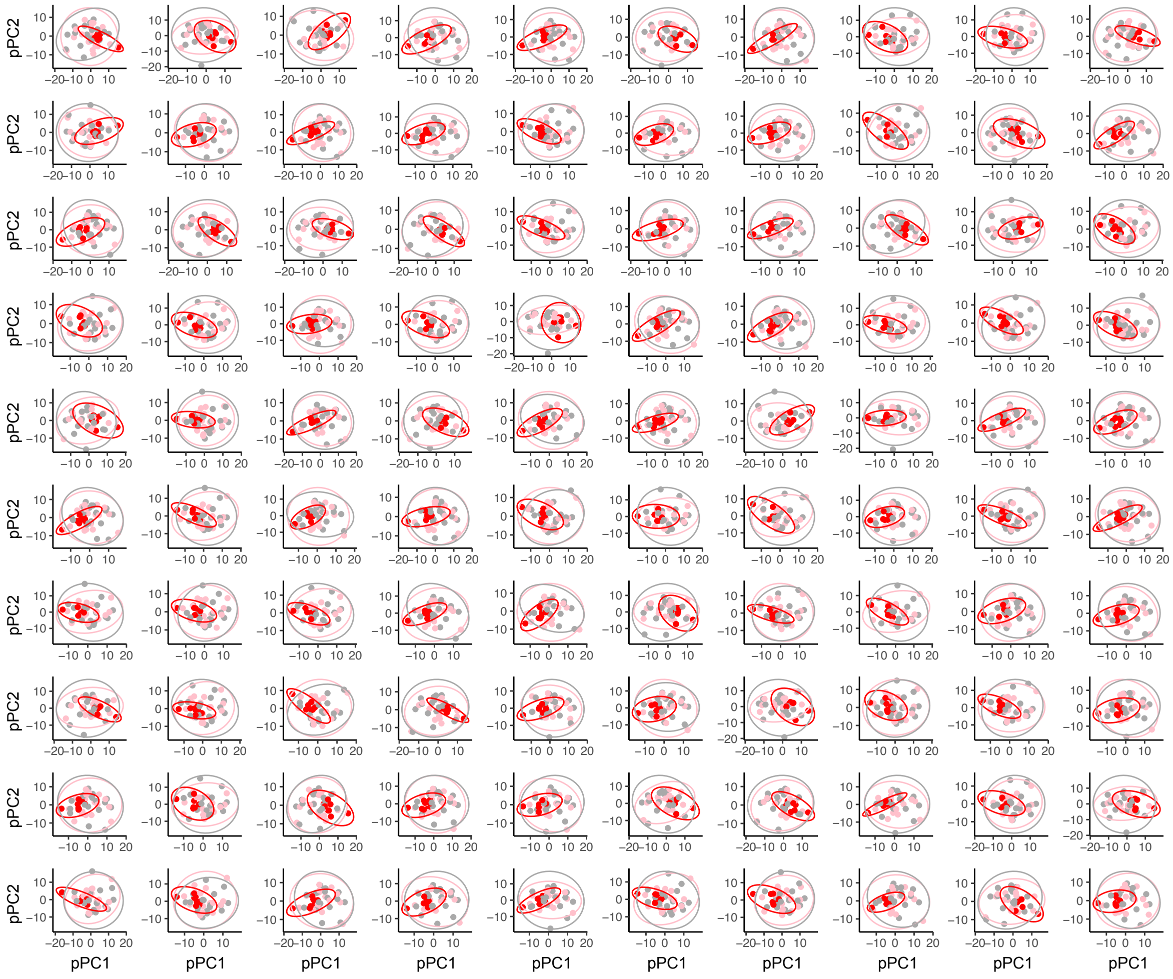

Supplementary Figure 7
Overlay of known ploidy (diploid, diploid/polyploid, and polyploid) of each species on pPC1 and pPC2. Diploid and diploid/polyploid species tend to have positive values of pPC1 whereas polyploids span the entire range of pPC1 and pPC2.

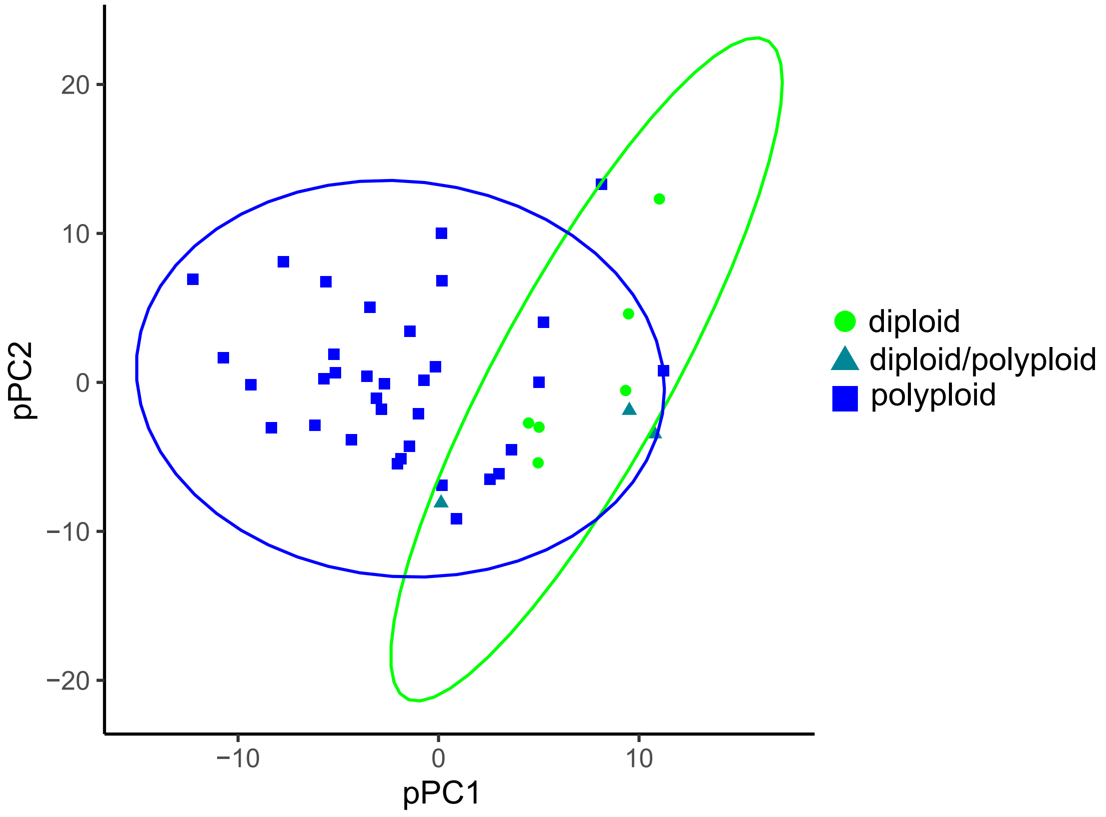

Supplementary Figure 8

Phenotypic disparity in pairwise species comparisons was only explained by floral color (pPC1: $F_{(5,808)} = 2.42$, $p = 0.034$; pPC2: $F_{(5,808)} = 3.88$, $p = 0.0018$), and not by sympatry/allopatry (pPC1: $F_{(5,808)} = 1.49$, $p = 0.222$; pPC2: $F_{(5,808)} = 0.189$, $p = 0.664$). Red-red species pair comparisons always had the lowest pairwise phenotypic disparity.

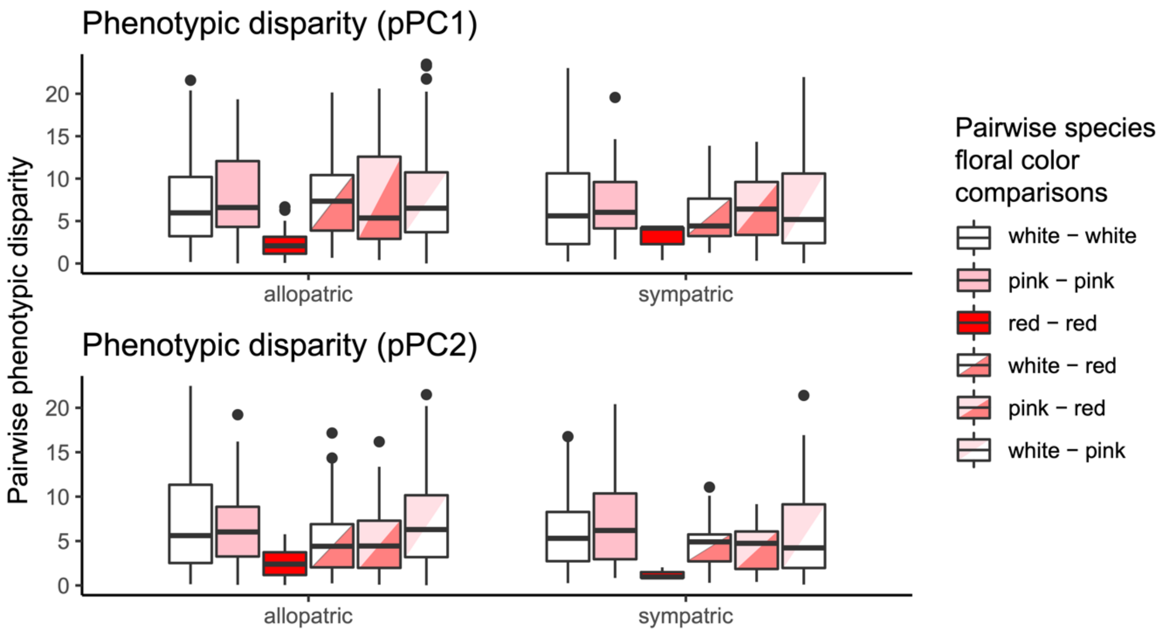

Supplementary Table 1.
Comparison of likelihood models tested for floral color transitions in the ancestral state reconstruction analysis, with log-likelihood (lnLik) and Akaike Information Criterion (AIC) scores. The lowest AIC scores are in bold. Character states: 0 = pink, 1 = red, 2 = white. The transition rate of pink to red flowers is represented by q01, for example.

**Model tested**	**Constraints**	**Free parameters**	**lnLik**	**AIC**
All rates different	–	6: q_{01}, q_{10}, q_{02}, q_{20}, q_{12}, q_{21}	−43.065	98.130
Equal rates	$q_{01} = q_{10} = q_{02} = q_{20} = q_{12} = q_{21}$	1: q	−45.989	**93.977**
Symmetric rates	$q_{01} = q_{10}$, $q_{02} = q_{20}$, $q_{21} = q_{12}$	3: q_{01}, q_{02}, q_{12}	−45.662	97.324
Stepwise reversible	$q_{02} = 0$, $q_{20} = 0$<br><br>$q_{12} = 0$, $q_{21} = 0$	4: q_{01}, q_{10}, q_{12}, q_{21}<br>4: q_{01}, q_{10}, q_{02}, q_{20}	−43.383	**94.765**
Stepwise pink-red (01) irreversible	$q_{02} = 0$, $q_{20} = 0$, $q_{10} = 0$	3: q_{01}, q_{12}, q_{21}	−63.444	132.888
Stepwise red-white (12) irreversible	$q_{02} = 0$, $q_{20} = 0$, $q_{21} = 0$	3: q_{01}, q_{12}, q_{10}	−49.619	105.238
Stepwise red-pink (10) and red-white (12) irreversible	$q_{10} = 0$, $q_{21} = 0$, $q_{12} = 0$	3: q_{01}, q_{02}, q_{20}	−54.378	114.755

Supplementary Table 2.
Summaries for the two best models for ancestral state reconstruction: equal rates and stepwise reversible models. Transition rates were estimated using maximum likelihood analysis with the ace function from the R ape package. MT = percentage mean total time spent in each state, TC = average number of total changes, C = number of changes per transition. Character states: 0 = pink (p), 1 = red (r), 2 = white (w). Most frequent transitions are in bold.

**Model**	**Free para-meters**	**Parameter estimates**	**MT**	**TC**	**Transition**	**C**
ER	q	q = 0.8895892	0/pink = 36.0%<br>1/red = 30.4%<br>2/white = 33.6%	174.583	0 -> 1 p -> r<br>**0 -> 2 p -> w**<br>1 -> 0 r -> p<br>1 -> 2 r -> w<br>2 -> 0 w -> p<br>2 -> 1 w -> r	29.134<br>31.08<br>28.916<br>29.468<br>29.194<br>26.791
Stepwise reversible corrected	q_{01}, q_{10}, q_{02}, q_{20}	$q_{01} = 11.953$<br>$q_{10} = 29.690$<br>$q_{02} = 3.753$<br>$q_{20} = 3.353$	0/pink = 40.1%<br>1/red = 16.2%<br>2/white = 43.6%	1043.805	**0 -> 1 p -> r**<br>0 -> 2 p -> w<br>**1 -> 0 r -> p**<br>1 -> 2 r -> w<br>2 -> 0 w -> p<br>2 -> 1 w -> r	398.191<br>125.215<br>399.128<br>0<br>121.271<br>0

Supplementary Table 3. Spearman correlation coefficient for the total species dataset in support of Figure 2. Standard Spearman's r values appear above the diagonal and phylogenetically corrected Spearman's r values appear below the diagonal. Correlation coefficients with values in bold if $P < 0.05$ after correction for multiple comparisons. Floral color integer values are white = 0, pink = 1, red = 2. Floral tube extension and reproductive organ exsertion were coded as (-1, 0, 1/below, equal to, or above calyx or corolla, respectively). Inflorescence class was coded as (1, 2, 3 for one flower, few flowers, or many flowers).

	Tube ext.	Repr. organ exs.	Calyx height	Calyx width	Petal limb length	Inflor. class	Petal color
Tube extension	--	**0.37**	0.07	0.09	0.22	0.16	-0.05
Reproductive organ exsertion	0.38	--	0.22	-0.05	0.18	0.12	0.11
Calyx height	0.07	0.16	--	**0.33**	**0.56**	0	**0.41**
Calyx width	0.10	-0.17	0.09	--	**0.32**	-0.1	0.01
Petal limb length	0.34	0.10	0.47	0.12	--	0.03	**0.37**
Inflorescence class	0.16	0.08	0.04	-0.24	0.02	--	-0.19
Petal color	0.07	0.16	0.35	-0.11	0.19	-0.13	--

Supplementary Table 4. Phylogenetic PCA loadings. Traits that are categorical in nature were assigned numerical values as designated. The ITS gene tree was used for pPCA with 43 out of 47 species (four did not have ITS sequences publicly available). The phylogenetic signal (λ) estimated for the dataset was 7.56e-05.

	**PC1**	**PC2**	**PC3**	**PC4**	**PC5**	**PC6**
**Floral tube extension from calyx (-1, 0, 1)**	-0.6872	-0.1749	0.5245	0.2895	-0.1871	0.3213
**Reproductive organ exsertion from corolla (-1, 0, 1)**	-0.5512	-0.4468	0.3607	-0.4707	0.3010	-0.2329
**Calyx height**	-0.6324	0.3016	-0.5346	-0.2362	0.3008	0.2777
**Calyx width**	-0.0847	0.7487	0.3579	0.3534	0.4013	-0.1351
**Petal limb length**	-0.7519	0.3265	-0.2302	0.0632	-0.4196	-0.3082
**Inflorescence class (1, 2, 3 / one, few, many)**	-0.2172	-0.6129	-0.3612	0.6097	0.2486	-0.1145
**Proportion of Variance**	0.2993	0.2273	0.1671	0.1436	0.1025	0.0602
**Cumulative Proportion**	0.2993	0.5266	0.6937	0.8373	0.9398	1.0000

Supplementary Table 5. Genotypic and phenotypic information. Genbank accessions for sequences used in ITS gene tree. Numbers after the species name were assigned arbitrarily and indicate alternate copies as represented in Popp and Oxelman 2007 or Mesler *et al*. 2021, and stars (*) indicate that only a partial ITS sequence was available. Phenotypic measurement sources are included when data was not taken or measured from the Flora of North America or the Jepson eFlora. Herbarium specimen IDs are given for the Harvard University Herbaria (HUH) collections. Field measurements were taken in June 2021. Websites were visited from May 2021 to December 2021.

**Species**	**Genbank Accession**	***Silene* subgenus and section, according to Jafari et al. 2020**	**Phenotypic measurement source other than the Flora of North America**
S. acaulis	KJ918487.1	subg. *Silene* sect. *Siphonomorpha*	Petal limb length; Montana Field Guide https://fieldguide.mt.gov
S. antirrhina	DQ908631.1	subg. *Silene* sect. *Sclerophyllae*	Stem height; University of Washington Burke Herbarium https://biology.burke.washington.edu/herbarium/imagecollection/taxon.php?Taxon=Silene%20antirrhina
S. bernardina	DQ908632.1	subg. *Behenantha* sect. *Physolychnis*	
S. bernardina 2	DQ908633.1	subg. *Behenantha* sect. *Physolychnis*	
S. bolanderi	MN231219.1	subg. *Behenantha* sect. *Physolychnis*	All measurements taken from 3 individuals (up to 10 flowers) in the field, June 2021
S. campanulata ssp. *glandulosa 1*	DQ908634.1	subg. *Behenantha* sect. *Physolychnis*	Leaf length and petal limb length measurements taken from 3 individuals (up to 10 flowers) in the field, June 2021
S. campanulata ssp. *glandulosa 2*	DQ908635.1	subg. *Behenantha* sect. *Physolychnis*	See above *S. campanulata* ssp. *glandulosa 1*
S. caroliniana ssp. *caroliniana*	DQ908636.1	subg. *Behenantha* sect. *Physolychnis*	
S. caroliniana ssp. *pensylvanica 1*	DQ908637.1	subg. *Behenantha* sect. *Physolychnis*	
S. caroliniana ssp. *pensylvanica 2*	DQ908638.1	subg. *Behenantha* sect. *Physolychnis*	
S. caroliniana ssp. *wherryi*	DQ908639.1	subg. *Behenantha* sect. *Physolychnis*	
S. douglasii ssp. *oraria*	KX757366.1	subg. *Behenantha* sect. *Physolychnis*	
S. drummondii	MG235595.1	subg. *Behenantha* sect. *Physolychnis*	Stem length; Gleason and Cronquist (1991)
S. hitchguirei	MG235315.1*	subg. *Behenantha* sect. *Physolychnis*	Calyx width; median calculated from 10 flowers across four specimens mounted on HUH01752320 and HUH00549615
S. hookeri 1	MN231227.1	subg. *Behenantha* sect. *Physolychnis*	
S. hookeri 2	MN231225.1	subg. *Behenantha* sect. *Physolychnis*	
S. hookeri ssp. *hookeri*	DQ908641.1	subg. *Behenantha* sect. *Physolychnis*	

S. invisa	DQ908642.1	subg. *Behenantha* sect. *Physolychnis*	
S. involucrata	KX757387.1*	subg. *Behenantha* sect. *Physolychnis*	Calyx width; https://nature.ca/aaflora/data/www/casiin.htm
S. laciniata ssp. *californica*	DQ908644.1	subg. *Behenantha* sect. *Physolychnis*	
S. laciniata ssp. *greggii*	DQ908645.1	subg. *Behenantha* sect. *Physolychnis*	
S. laciniata ssp. *laciniata*	DQ908646.1	subg. *Behenantha* sect. *Physolychnis*	
S. lemmonii 1	DQ908648.1	subg. *Behenantha* sect. *Physolychnis*	
S. lemmonii 2	DQ908649.1	subg. *Behenantha* sect. *Physolychnis*	
S. menziesii 1	DQ908651.1	subg. *Silene* sect. *Anotites*	
S. menziesii 2	MN231234.1	subg. *Silene* sect. *Anotites*	
S. menziesii 3	DQ908652.1	subg. *Silene* sect. *Anotites*	
S. nelsonii	MN231215.1	subg. *Behenantha* sect. *Physolychnis*	
S. nivea	DQ908654.1	subg. *Behenantha*, incertae sedis	Leaf length; https://dnr.wi.gov/topic/EndangeredResources/Plants.asp?mode=detail&SpecCode=PDCAR0U120
S. nutans	DQ908655.1	subg. *Silene* sect. *Siphonomorpha*	Not native to North America, used for ancestral state reconstruction as second outgroup
S. occidentalis	DQ908656.1	subg. *Behenantha* sect. *Physolychnis*	
S. oregana	DQ908657.1	subg. *Behenantha* sect. *Physolychnis*	
S. ostenfeldii	MG234678.1*	subg. *Behenantha* sect. *Physolychnis*	Petal limb length; median calculated from five flowers from specimen HUH01753078
S. ovata	DQ908658	subg. *Behenantha* sect. *Physolychnis*	
S. parishii	AJ629910.1	subg. *Behenantha* sect. *Physolychnis*	
S. parryi	DQ908659.1	subg. *Behenantha* sect. *Physolychnis*	
S. petersonii	X86886.1	subg. *Behenantha* sect. *Physolychnis*	
S. polypetala	DQ908660.1	subg. *Behenantha* sect. *Physolychnis*	
S. regia	AY116476.1	subg. *Behenantha* sect. *Physolychnis*	
S. repens 1	DQ908661.1	subg. *Silene* sect. *Auriculatae*	Flora of China; leaf length http://www.efloras.org/florataxon.aspx?flora_id=2&taxon_id=200007065
S. repens 2	DQ908662.1	subg. *Silene* sect. *Auriculatae*	See above *S. repens 1*
S. repens 3	DQ908663.1	subg. *Silene* sect. *Auriculatae*	See above *S. repens 1*
S. rotundifolia	AY116477.1	subg. *Behenantha* sect. *Physolychnis*	

S. salmonacea	MN231233.1	subg. *Behenantha* sect. *Physolychnis*	All measurements taken from 3 individuals (up to 10 flowers) in the field, June 2021
S. scaposa	DQ908664.1	subg. *Behenantha* sect. *Physolychnis*	
S. scouleri ssp. *scouleri*	DQ908665.1	subg. *Behenantha* sect. *Physolychnis*	
S. seelyi	DQ908666.1	subg. *Silene* sect. *Anotites*	
S. serpentinicola	MN231232.1	subg. *Behenantha* sect. *Physolychnis*	
S. sorensenis	MG237323.1*	subg. *Behenantha* sect. *Physolychnis*	
S. stellata 1	DQ908667.1	subg. *Behenantha* sect. *Physolychnis*	
S. stellata 2	DQ908668.1	subg. *Behenantha* sect. *Physolychnis*	
S. subciliata	DQ908669.1	subg. *Behenantha* sect. *Physolychnis*	
S. suksdorfii 1	DQ908670.1	subg. *Behenantha* sect. *Physolychnis*	
S. suksdorfii 2	DQ908671.1	subg. *Behenantha* sect. *Physolychnis*	
S. uralensis	KM011961.2	subg. *Behenantha* sect. *Physolychnis*	
S. verecunda	DQ908673.1	subg. *Behenantha* sect. *Physolychnis*	
S. verecunda ssp. *andersonii*	DQ908630.1	subg. *Behenantha* sect. *Physolychnis*	
S. virginica	DQ908674.1	subg. *Behenantha* sect. *Physolychnis*	
S. williamsii	DQ908675.1	subg. *Silene* sect. *Anotites*	
Saponaria officinalis	MF401078.1	outgroup	All measurements from the Flora of China; http://www.efloras.org/florataxon.aspx?flora_id=2&taxon_id=220011957
